# Mps1 kinase regulates tumor cell viability via its novel role in mitochondria

**DOI:** 10.1038/cddis.2016.193

**Published:** 2016-07-07

**Authors:** X Zhang, Y Ling, Y Guo, Y Bai, X Shi, F Gong, P Tan, Y Zhang, C Wei, X He, A Ramirez, X Liu, C Cao, H Zhong, Q Xu, R Z Ma

**Affiliations:** 1 State Key Laboratory for Molecular Developmental Biology, Institute of Genetics and Developmental Biology, Chinese Academy of Sciences, Beijing 100101, China; 2 Beijing Institute of Biotechnology, Beijing 100850, China; 3 Graduate School, University of the Chinese Academy of Sciences, Beijing 100149, China; 4 University of Colorado at Boulder, Boulder, CO 80302, USA

## Abstract

Targeting mitotic kinase monopolar spindle 1 (Mps1) for tumor therapy has been investigated for many years. Although it was suggested that Mps1 regulates cell viability through its role in spindle assembly checkpoint (SAC), the underlying mechanism remains less defined. In an endeavor to reveal the role of high levels of mitotic kinase Mps1 in the development of colon cancer, we unexpectedly found the amount of Mps1 required for cell survival far exceeds that of maintaining SAC in aneuploid cell lines. This suggests that other functions of Mps1 besides SAC are also employed to maintain cell viability. Mps1 regulates cell viability independent of its role in cytokinesis as the genetic depletion of Mps1 spanning from metaphase to cytokinesis affects neither cytokinesis nor cell viability. Furthermore, we developed a single-cycle inhibition strategy that allows disruption of Mps1 function only in mitosis. Using this strategy, we found the functions of Mps1 in mitosis are vital for cell viability as short-term treatment of mitotic colon cancer cell lines with Mps1 inhibitors is sufficient to cause cell death. Interestingly, Mps1 inhibitors synergize with microtubule depolymerizing drug in promoting polyploidization but not in tumor cell growth inhibition. Finally, we found that Mps1 can be recruited to mitochondria by binding to voltage-dependent anion channel 1 (VDAC1) via its C-terminal fragment. This interaction is essential for cell viability as Mps1 mutant defective for interaction fails to main cell viability, causing the release of cytochrome *c*. Meanwhile, deprivation of VDAC1 can make tumor cells refractory to loss of Mps1-induced cell death. Collectively, we conclude that inhibition of the novel mitochondrial function Mps1 is sufficient to kill tumor cells.

Massive chromosome missegregation induces cell death as observed by Theodor Boveri in the early 1900s.^1^ However, the underlying mechanism remains elusive. The spindle assembly checkpoint (SAC) is a dominant machine monitoring chromosomal segregation during mitosis by delaying the onset of anaphase until all chromosomes are properly captured by microtubules. The SAC consists of kinetochore association sensors, including Mps1 (monopolar spindle 1), Bub1 (budding uninhibited by benzimidazole 1 homolog) and Aurora B; a signaling transducer termed the mitotic checkpoint complex (MCC), including CDC20 (cell division cycle 20), BubR1 (Bub1-related kinase), Bub3 (budding uninhibited by benzimidazole 3 homolog) and Mad2 (mitotic arrest deficient-like 2); and an effector APC/C (anaphase-promoting complex/cyclosome) that is inhibited by MCC in response to an active SAC.^2^ Loss of SAC by inactivation of checkpoint sensors or signaling transducers elicits massive chromosome missegregation, induces severe gain or loss of chromosomes and eventually causes cell death.^3, 4, 5, 6^ Meanwhile, a weakened SAC due to the haploinsufficiency of the checkpoint proteins Mad1, Mad2, Bub1, BubR1 and CENP-E (centromere protein E) does not cause cell death but facilitates tumorigenesis.^7, 8, 9, 10, 11^ These studies suggest that the fate of these cells is dependent on their respective degree of SAC deficiency. Notably, in these studies SAC proteins were constitutively disturbed, raising the possibility that other signaling pathways could be affected as SAC proteins have functions beyond SAC regulation.^12, 13, 14^

Mps1 is an essential component of SAC that senses SAC signal by promoting MCC formation via kinetochore recruitment of Mad2, CENP-E and Knl1 (kinetochore-null protein 1).^15, 16, 17, 18, 19^ Recent studies show that Mps1 can discriminate between on or off SAC signaling by binding to NDC80c via the motif that associates microtubules.^20, 21^ Following SAC, Mps1 is involved in regulating chromosome alignment by phosphorylating Borealin, a component of chromosomal passenger complex (CPC).^22, 23^ In addition, Mps1 plays multiple roles beyond mitosis, including centrosome duplication, cytokinesis, ciliogenesis and DNA damage response.^18, 24, 25, 26, 27, 28^ Mps1 is indispensable for cell survival as loss of Mps1 function by specific siRNA or Mps1 kinase inhibitors causes significant cell death; it has been proposed that Mps1 regulates this process through its roles in SAC.^29, 30, 31^

Mps1 kinase is overexpressed in a variety of tumor types.^32, 33, 34, 35^ In breast cancer, high levels of Mps1 correlate with tumor grades; reducing Mps1 level induces massive apoptosis but allows a selective survival of tumor cells with less aneuploidy.^32^ Our recent results in colon cancer cells showed that overexpression of Mps1 facilitate the survival of tumor cells with higher aneuploidy by decreasing SAC threshold.^35^ To further uncover the roles of high levels of Mps1 in tumorigenesis, we examined Mps1 levels in various stages of colon cancer tissues and found that Mps1 level peaks in tissues at stage II, at which stage tumor cells encounter various survival stresses, including genome instability. Aneuploid colon cancer cell lines bear higher levels of Mps1 than diploid cell lines and the amount of Mps1 required for cell survival is far more than that of maintaining SAC, suggesting that other functions of Mps1 are also employed to maintain cell viability. Short-term inhibition of Mps1 activity in mitosis with inhibitors at a dose of more than SAC depletion is sufficient to cause dividing cell death and increase mitochondrial fragmentation simultaneously. Finally, we found that Mps1 can regulate the release of cytochrome *c* by associating with mitochondrial protein VDAC1 (voltage-dependent anion channel 1). Based on these findings, we postulated that high levels of Mps1 contribute to survival of aneuploid cancer cells via its roles in SAC and mitochondria.

## Results

### High levels of Mps1 contribute to survival of aneuploid tumor cells

**Figure 1:**
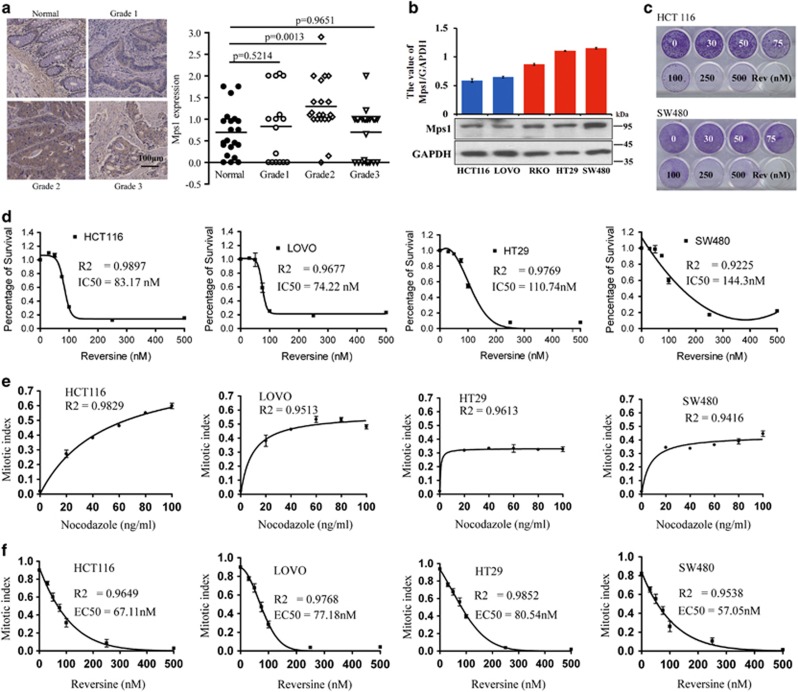
High levels of Mps1 attribute to the survival of aneuploid tumor cells. (**a**) The Mps1 level of 96 colon cancer tissues from 48 subjects with clinical stages ranging from I to III. The slides were treated following a standard protocol and stained with an anti-Mps1 antibody in a dilution of 1 : 100. The quantification and statistical results of the amount of Mps1 are presented. (**b**) The quantification results of the amount of Mps1 in five cell lines. (**c** and **d**) Four colorectal cancer cell lines, including HCT116, SW480, LoVo and HT29 (the result of LoVo and HT29 was put in Supplementary Figure 1f), were treated with the Mps1 inhibitor Reversine at escalating doses and the cell viability was determined via crystal violet staining. Data are representative of three independent experiments. Error bars, S.D. (**e**) The dose of Nocodazole required for the mitotic checkpoint was establish in four colorectal cancer cell lines by counting the mitotic index. Data are representative of three independent experiments. Error bars, S.D. (**f**) The dose of Reversine required for the deletion of the spindle assembly checkpoint in four cell lines triggered by Nocodazole was determined by counting the mitotic index. The mitotic cells were collected by shaking off the Nocodazole-treated cells and were co-incubated with Reversine using the indicated doses. Data are representative of three independent experiments. Error bars, S.D.

Mps1 is overexpressed in a variety of tumor types (Supplementary Figure 1a).^32, 33, 34, 35^ Consistently, we confirmed that the protein levels of Mps1 are also significantly higher in the colon cancer tissue than the adjacent and normal tissues from 18 subjects (Supplementary Figures 1b and c). Next, we examined the Mps1 level in 96 colon cancer tissues from 48 subjects with clinical stages ranging from I to III and found that Mps1 levels are significantly higher in the stage II carcinoma (Figure 1a and Supplementary Figure 1d). This suggests that the progressive tumor cells before metastasis are highly addicted to Mps1 proteins.

The colon cancer cells in development are characterized with increasing genome instability because of genetic and epigenetic alterations.^36, 37^ We analyzed four validated colorectal cancer cell lines, including two near diploid lines (HCT116 and LoVo) and two aneuploid lines (HT29 and SW480) (Supplementary Figure 1e). As shown, Mps1 is overexpressed in the two aneuploid lines compared with the other two diploid lines (Figure 1b). This result is consistent with the finding in breast cancer.^32^ Inhibition of Mps1 kinase activity by Reversine, a specific Mps1 kinase inhibitor, triggers cell death in both diploid and aneuploid tumor cells in a dose-dependent manner (Figure 1c and Supplementary Figure 1f). Interestingly, more Reversine is required to inhibit the growth of SW480 and HT29 than HCT116 and LoVo, and this is consistent with the fact that aneuploid cell line bears higher levels of Mps1. Given that SAC has been proven to be essential for cell viability, ^5, 6^ we set to determine whether high levels of Mps1 contribute to cell survival by modulating SAC. All examined cell lines have a robust SAC that can be ignited by the microtubule toxin Nocodazole and then be extinguished by a specific Mps1 inhibitor, Reversine (Figures 1e and f).^38^ The doses of Reversine required to kill cells *versus* that required for SAC depletion are equivalent in diploid cells, but much higher in aneuploid cells (Figures 1d and f). These findings suggest that in aneuploid tumor cells high levels of Mps1 regulate cell viability by employing its roles not only in SAC but also in other pathways uncovered.

### Mps1 promotes tumor cell survival independent of its role in cytokinesis

**Figure 2:**
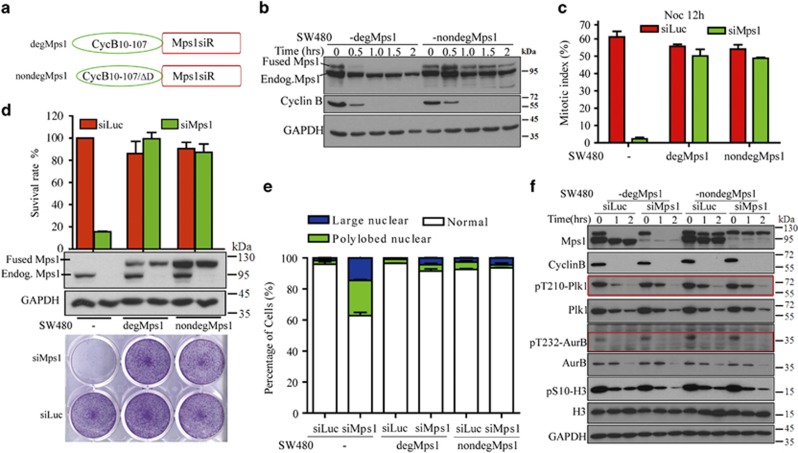
High levels of Mps1 promote tumor cells survival independent of its role in cytokinesis. (**a**) Mps1 was fused to the wild-type or mutated N-terminus of Cyclin B1, allowing Mps1 degradation after metaphase (degMps1) or degradation resistance through mitosis (undegMps1). (**b**) Stable cell lines SW480-degMps1 and SW480-undegMps1 were released from Nocodazole and subjected to western blotting with antibodies at the indicated time points. (**c**) Cells SW480-degMps1 and SW480-undegMps1 were treated with thymidine for 24 h and then released into medium with Nocodazole for 12 h followed by counting of the mitotic index. Data are representative of three independent experiments. Error bars, S.D. (**d**) Equal numbers of SW480-degMps1 and SW480-undegMps1 cells were transfected twice with siRNA against Mps1 and after 6 days the cell viabilities were determined by crystal violet staining. The statistical results of the surviving cells are shown in the upper panel and the siRNA efficiency is shown in the middle panel. Data are representative of three independent experiments. Error bars, S.D. (**e**) SW480-degMps1 and SW480-undegMps1 were transfected with siRNA as stated in (**d**) and the cells were counterstained with DAPI; the statistical results were obtained by counting more than 300 cells and plotted using Graphpad software (La Jolla, CA, USA). Data are representative of three independent experiments. Error bars, S.D. (**f**) Cells SW480-degMps1 and SW480-undegMps were transfected with siRNA as stated in (**d**) and the cells were subjected to western blotting with antibodies as indicated

Mps1 has been suggested to be involved in cytokinesis.^24, 25^ To further investigate whether cytokinesis failure by Mps1 inactivation is involved in cell viability, we developed a cell cycle-dependent degradable Mps1 by fusing it to the N-terminal fragment of Cyclin B (10–107 aa) that mediates Cyclin B degradation at the onset of anaphase.^39, 40^ As a control, Mps1 is also fused to the same fragment, but with a deletion of D-box motif that is essential for Cyclin B degradation.^41^ These two fusion proteins, termed degradable Mps1 (degMps1) and nondegradable Mps1 (nondegMps1) respectively, were stably expressed in SW480 cells (Figure 2a). As expected, degMps1 showed a degradation pattern similar to that of wild-type Cyclin B, whereas the nondegMps1 level fluctuates similarly to wild-type Mps1 (Figure 2b). While tracing Mps1 levels in Nocodazole-released cells, we found that the depletion of degMps1 persists for at least 3 h after prometaphase (Supplementary Figure 2a). Using this model, we found that Mps1 depletion after metaphase affects neither SAC establishment nor mitotic progression (Figure 2c and Supplementary Figure 2b), although the level of forced expressed of Mps1 is less than the endogenous version. SW480-degMps1 and SW480-nondegMps1 cells have a similar survival ratio compared with wild-type SW480 cells treated with Mps1-specific siRNA (Figure 2d). No difference in cell viability was observed between SW480-degMps1 and SW480-nondegMps1 when these cell lines were treated with siMps1 for 48 h, followed by NMS-P715 at indicated doses for additional 7 days (Supplementary Figure 2c), suggesting that no difference of kinase activity existed between the two versions of fusion Mps1. We also determined the karyotypes of SW480-degMps1 and SW480-nondegMps1 upon the depletion of endogenous Mps1 by siMps1. The ratio of the aberrant cells, including the large nuclear and polylobed cells, is not significantly different between SW480-degMps1 and SW480-nondegMps1 (Figure 2e).

Next, we tested the activity of Aurora B and Plk1 (polo-like kinase 1), both of which have established roles in cytokinesis. We found that the kinase activity of Plk1 and Aurora B increases significantly in SW480-degMps1 upon loss of endogenous Mps1 (Figure 2f and Supplementary Figure 2d), suggesting that the function of Mps1 in cytokinesis is compensated by other kinases. This supports that high levels of Mps1 promotes tumor cell survival independent of its role in cytokinesis.

### Single-cycle inhibition of Mps1 in mitosis is sufficient to cause cell death

**Figure 3:**
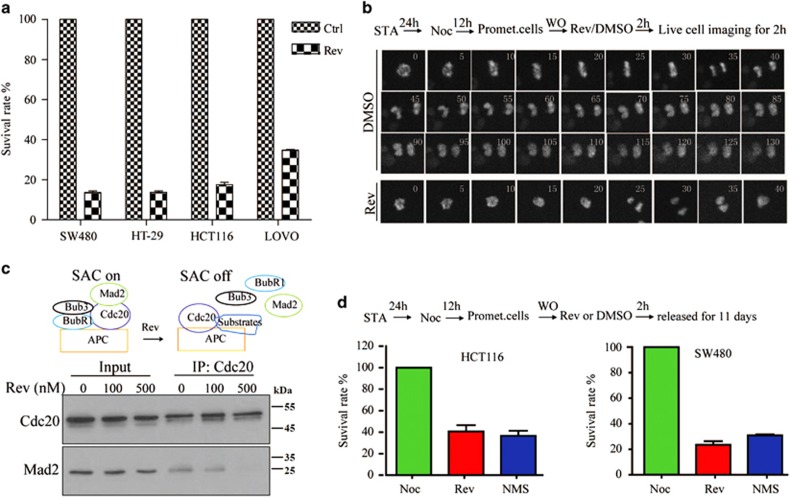
Single-cycle loss of SAC by temporary Mps1 inhibition is sufficient to cause cell death. (**a**) Four colorectal cancer cell lines, including HCT116, LoVo, HT29 and SW480, were treated with the Mps1 inhibitor Reversine at escalating doses for 24 h and the cell viability was determined via crystal violet staining. Data are representative of three independent experiments. Error bars, S.D. (**b**) Mitotic cells released from Nocodazole exit mitosis without SAC and chromosome alignment. Mitotic SW480 cells were treated as indicated and the cell division process was traced via live cell imaging. (**c**) Mitotic SW480 cells were treated with Reversine at the indicated concentrations for 24 h and subjected to immunoprecipitation analysis with antibodies as shown. (**d**) The 2-h inhibition of Mps1 by two specific Mps1 inhibitors can exclusively exert a significant inhibitory effect on cell growth. Data are representative of three independent experiments. Error bars, S.D.

Genetic and pharmaceutical inhibition of Mps1 causes cell death in several tumors cell lines.^6, 34, 42^ However, in these studies Mps1 functions were constitutively disturbed for several days with either Mps1 inhibitors or siRNA, raising the question that other functions of Mps1 beyond SAC could be also affected. It has been reported that short-term inhibition of Mps1 for 2 days is sufficient to cause cell death.^43^ Consistently, we found that treatment of tumor cells with Reversine or NMS-P715, another Mps1 inhibitor,^30^ for 24 h yielded similar result (Figure 3a and Supplementary Figure 3a). To confirm that Mps1 regulates cell viability through its role in SAC, we set up a procedure allowing SAC depletion only once in a single mitotic process, termed as single-cycle inhibition. Briefly, Nocodazole-treated SW480 cells were shaken off and released into Reversine for 2 h, and then subject to live cell imaging in fresh medium (Figure 3b). A 2-h treatment can limit Mps1 inhibition only in mitosis. First, this treatment duration is long enough for the majority of SW480 cells to pass through mitosis in the presence of Reversine or DMSO (Supplementary Figure 3b). Second, Mps1 kinase activity can be completely disturbed with this procedure, including the chromosomes alignment and SAC. As shown, no metaphase plate formed in all examined cells under this procedure (Figure 3b). Consistent with this, 2-h treatment of Reversine in mitosis also turns off SAC, evidenced by the dissociation of Mad2 with Cdc20, a hallmark of the inactivation of SAC signal (Figure 3c). Based on these findings, we tested whether single-cycle inhibition of Mps1 affected cell viability. As shown in Figure 3d, 2-h inhibition of Mps1 can exclusively exert a significant inhibitory effect on cell viability. Collectively, we conclude that deprivation of Mps1 mitotic function through a single-cycle inhibition procedure is sufficient to cause tumor cell death.

### Single-cycle inhibition of Mps1 kills tumor cells independent of the polyloidization process

**Figure 4:**
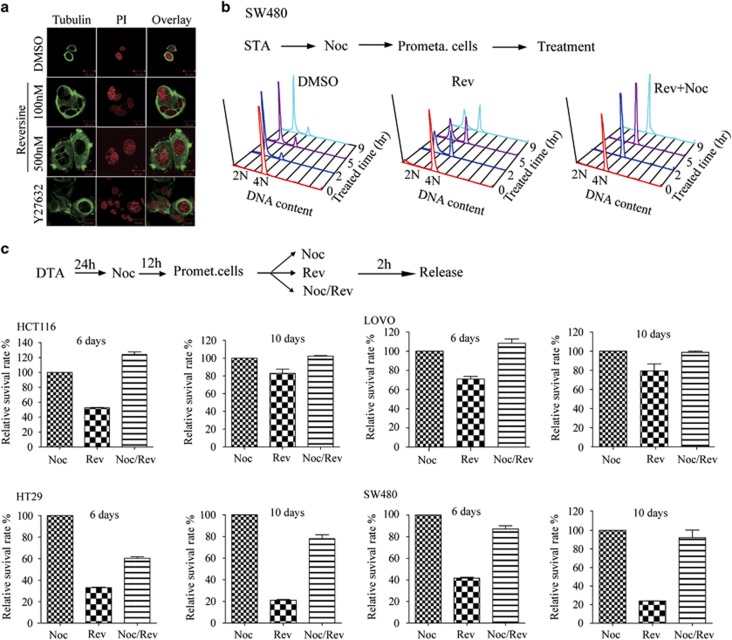
Loss of SAC by Mps1 inhibition preferentially kills dividing tumor cells. (**a**) SW480 cells were treated with Reversine at the indicated doses for 5 days and stained with anti-*α*-tubulin and PI. Y27636 is an inhibitor of Rho-kinase and can cause cytokinesis failure. (**b**) SW480 cells were arrested at prometaphase by a sequential single thymidine and Nocodazole treatment procedure; mitotic cells were collected and released into the fresh medium with compounds as shown and treated for the indicated amounts of time before flow cytometry analysis. STA, single thymidine arrest. (**c**) SW480 cells were synchronized by a sequential double thymidine and released as in Nocodazole for 12 h and then released and treated with drugs as indicated for 2 h. The cell viability was determined by crystal violet staining after 6 and 10 days. This experiment was repeated three times. Error bars, S.D. DTA, double thymidine arrest

Consistent with the previous finding, Mps1 inhibition for 24 or 48 h kills a large fraction of tumor cells but still leaves some cells alive; these remaining cells are polyploidy cells characterized with either large nuclear or multinuclear phenotypes (Figure 4a). The polyploidization could be either susceptible or refractory to cell death. To distinguish these two possibilities, we set to evaluate the relationship between polyploidy and cell viability. First, we investigate whether single-cycle inhibition of Mps1 affects cellular chromosome numbers by measuring the DNA content of mitotic cells. As shown, we found that single-cycle inhibition of Mps1 induced a partial polyploidization in SW480 cells (Figure 4b, middle panel); increased dosage but not extended duration of Mps1 inhibitor treatment increases the ratio of polyploidy (Figure 4b, middle panel, Supplementary Figure 4a). Notably, mitotic SW480 cells cotreated with both Nocodazole and Reversine all proceed without undergoing cytokinesis (Figure 4b, right panel). This suggests an accumulative effect of the checkpoint depletion and spindle disturbance affecting cytokinesis during the polyploidization process. The complete failure of cytokinesis is not due to the long-term effects of Reversine or Nocodazole, as the withdrawal of both drugs after 2 h of treatment yields the same result (Supplementary Figure 4b, middle panel).

Next, we investigated the relationship between cell polyploidization and viability by using the single-cycle Mps1 inhibition procedure. Consistently, single-cycle inhibition triggers cell death in all four colon cancer cell lines (Figure 4c). However, cotreatment with Reversine and Nocodazole yields a lower tumor death ratio compared with the tumor cells treated with only Reversine. A similar result was also achieved with Nocodazole and NMS-P715, another Mps1 inhibitor (Supplementary Figure 4c).^30^ Given that Reversine plus Nocodazole treatment causes nearly 100% tetraploidization (Figure 4b, right panel), we postulate that polyploid tumor cells are resistant to genome instability. To validate this finding, we also treated cells with Reversine and chemical BI 2536, a specific inhibitor of Plk1 that has an established role in cytokinesis.^44^ Consistently, cotreatment with BI 2536 and Reversine causes a higher polyploidization but shows a lower tumor cell death ratio compared with the BI 2536 alone (Supplementary Figures 4d and e). In addition, we analyzed the published data in which the fate of HCT116 cells was traced by live cell imaging in the presence of other Mps1 inhibitors.^45^ Consistent with our observation, the cell death ratio of the diploid cells is much higher than that of tetraploid cells in the presence of Mps1 inhibitors (Supplementary Figure 4f).

Based on these findings, we postulate that single-cycle inhibition of Mps1 kills tumor cells not by promoting polyploidization. These finding also suggests that there is no synergistic tumor killing effect among these chemicals that target the SAC and the spindle assembly process.

### Reduced Mps1 levels in tumor cells trigger apoptosis signaling pathway

**Figure 5:**
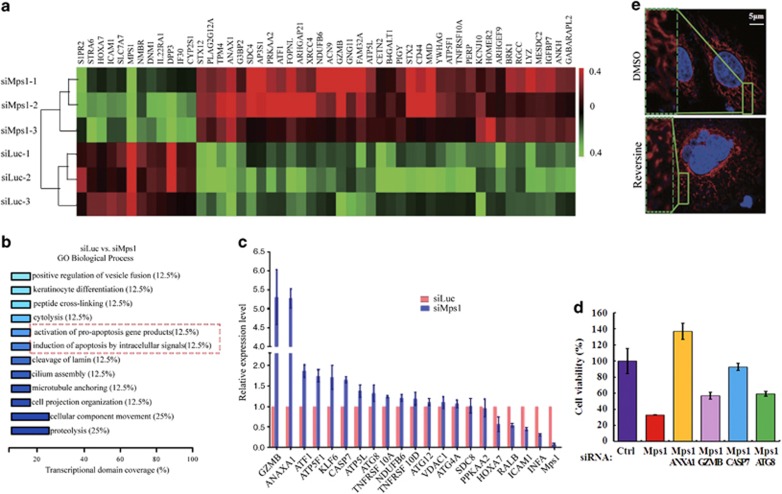
Decreased Mps1 levels in tumor cells trigger an apoptotic signaling pathway. (**a**) SW480 cells were transfected twice with the siRNA against Mps1 and after 72 h the mRNA were extracted, enriched and subjected to microarray analysis. The assay was repeated three times and the genes with 1.4-fold increase in expression were selected and clustered via MeV software. (**b**) GO analysis of affected genes upon perturbation of Mps1 functions via siRNA. (**c**) SW480 cells were transfected twice with Mps1 siRNA and after 72 h the cells were subjected to RNA extraction and qRT-PCR analysis with the primers against genes as indicated. Data are representative of three independent experiments. Error bars, S.D. (**d**) SW480 cells were transfected with indicated siRNA as indicated and kept in medium for 4 days. The cell viability was determined by crystal violet staining. Results are expressed as mean±S.E.M.; *P*<0.05 compared with siMps1 result. Data are representative of three independent experiments. Error bars, S.D. (**e**) SW480 cells were treated with DMSO or Reversine and then stained with Mitotracker (ThermoFisher Scientific, Grand Island, NY, USA) to observe the morphology of mitochondria

As mentioned above, the dose required for cell death is much higher than that for SAC depletion in aneuploid cells SW480 and HT29. To uncover the mechanism underlying high levels of Mps1 promoting cell survival, we determined the transcriptome of SW480 cells after Mps1 siRNA or Reversine. SW480 cells were treated with Mps1 siRNA or Reversine for 3 days and then total RNA was extracted and subjected to hybridization analysis via Affymetrix Human Transcriptome Arrays (HTA2) (Santa Clara, CA, USA). Mps1 depletion affects the transcriptional level of 57 genes, including 29 upregulated genes and 28 downregulated genes (Figure 5a). These 57 affected genes with the exception of Mps1 are also included in the gene list affected by Reversine. Gene Ontology (GO) and pathway analysis of the differentially expressed genes suggests that the apoptosis pathway has been triggered in the treated cells (Figure 5b and Supplementary Figure 5a).

The transcriptional changes of these apoptotic-related molecules were confirmed by quantitative PCR analysis (Figure 5c and Supplementary Figure 5b). Next, we determined whether reduction of these apoptotic genes related to Mps1 inhibition could avoid cell death. Among the upregulated genes, GZMB (granzyme B), Annexin A1 and Caspase 7 are well established for their roles in cell apoptosis. Reduction of these proteins via siRNAs decreases cell death induced by Mps1 knockdown at variable ratios (Figure 5d). ATG8 (autophagy-related 8), an ubiquitin-like protein essential for autophagosome formation,^46^ is also upregulated upon Mps1 depletion (Figure 5d). Knock down of ATG8 exerts a protective effect to a level comparable to GZMB. However, several inhibitors against the autophagy pathway failed to block Mps1 depletion-induced cell death (unpublished data).

Notably, several mitochondrial proteins related to apoptosis were enriched in GO analysis (Supplementary Figure 5c). Mitochondrial fission is an early event during apoptosis, occurring before caspase activation and membrane blebbing.^47^ We examined the morphology of mitochondria after a single-cycle inhibition and found that short-term Reversine treatment causes significant mitochondria fragmentation (Figure 5e) and increases the amount of cytochrome *c* in the cytoplasm of SW480 cells (Figure 7d). From above, we infer that high levels of Mps1 contribute to tumor cell survival by inhibiting the mitochondrial-dependent apoptotic signaling pathway.

### Mps1–VDAC1 interaction contributes to cell viability

**Figure 6:**
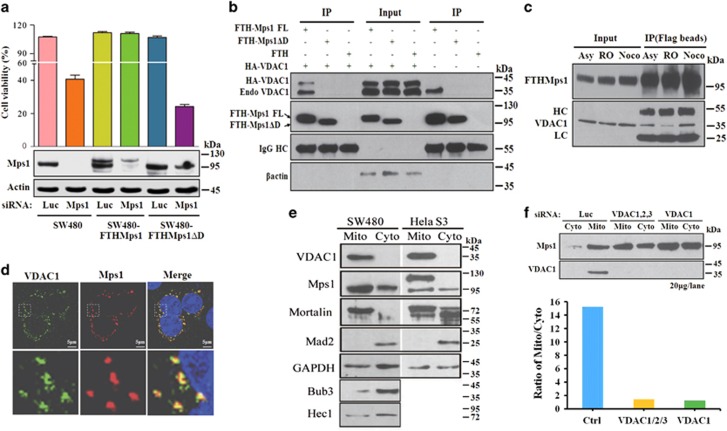
Mps1 interacts with VDAC1 and localizes on mitochondria. (**a**) SW480, stable cell lines SW480-FTHMps1 and SW480-FTHMps1ΔD were transfected with Mps1 siRNA and control siRNA. The cell viability was then determined after 7 days. Data are representative of three independent experiments. Error bars, S.D. (**b**) Retroviral vectors pRex-FTH-IRES-Hygro, pRex-FTHMps1-IRES-Hygro and pRex-FTHMps1ΔD-IRES-Hygro combined with pcDNA-HA-VDAC1 were co-transfected into 293T and then lysed after 48 h followed by immunoprecipitation and western blotting. (**c**) SW480-FTHMps1 cells, synchronized at G2/M (RO-33342, 9 *μ*M, 19 h) and prometaphase (Nocodazole 100 *μ*g/ml, 16 h, shaken-off) or asynchronized, were lysed and subjected to immunoprecipitation analysis. (**d**) SW480 cells were subjected to immunostaining with antibodies against Mps1 and VDAC1. (**e**) Mitochondria and cytoplasm of SW480 or Hela S3 were isolated and then subjected to western blot analysis with indicated antibodies. Per lane, 20 *μ*g of total protein was loaded. (**f**) SW480 cells were transfected with siRNA as indicated. The mitochondrial and cytoplasmic fractions were separated for western blotting and 20 *μ*g of total protein was loaded per lane. The distribution ratio of Mito/Cyto of Mps1 was measured

To further determine how Mps1 directly regulates apoptosis, we set to find the molecules that bind Mps1. Our previous results revealed that the C-terminal of Mps1 (792–857) is not required for autophosphorylation but essential for transphosphorylation of substrates and SAC activation.^48^ Following this, we found that the C-terminal is essential for Mps1 to maintain cell viability as substitution of endogenous Mps1 with a truncated Mps1 without its C-terminal (Mps1ΔD) is detrimental to SW480 cells (Figure 6a). To reveal the proteins of interest that bind to Mps1 via the C-terminal, an immuneprecipitation assay was performed. As a result, human VDAC1, a main mitochondrial outer membrane channel,^49^ was identified (Figure 6b,Supplementary Figure 6a and Supplementary Table 1). The association of Mps1 with VDAC1 is dominant during mitosis (Figure 6c). Furthermore, Mps1 colocalizes with VDAC1 with immunofluorescence staining (Figure 6d). Mps1 can also be detected in the purified mitochondrial fraction (Figure 6e). Interestingly, Mps1 is not the sole kinetochore protein residing in mitochondria. Kinetochore-binding proteins including Hec1 (basic helix-loop-helix (bHLH) DNA-binding superfamily protein HECATE 1) and Bub3, but not mad2, were also identified in the purified mitochondrial fraction, showing that communication networks may exist between the kinetochore and mitochondria. The amount of FTHMps1ΔD in mitochondria is significantly less than that of FTHMps1 (Supplementary Figure 6b). Treating cells with VDAC1 or VDACs siRNA can significantly reduce the amount of Mps1 in mitochondria (Figure 6f) as shown by immunofluorescence staining (Supplementary Figure 6c). Proteinase K digestion assay can efficiently eliminate Mps1 signal in the mitochondrial fraction (Supplementary Figure 6d), suggesting that Mps1 binds to VDAC1 via its N-terminal fragment that is exposed to cytoplasm.^50^ Neither the removal of C-terminal nor knock down of VDACs by siRNA treatment can completely abolish Mps1 in the mitochondrial fraction, suggesting that other proteins are also involved in mitochondrial recruitment of Mps1. A candidate protein Mortalin, a mitochondrial chaperon protein that binds Mps1 to regulate centrosome duplication,^51^ also partially colocalizes with Mps1 in mitochondria (Supplementary Figure 6e).

**Figure 7:**
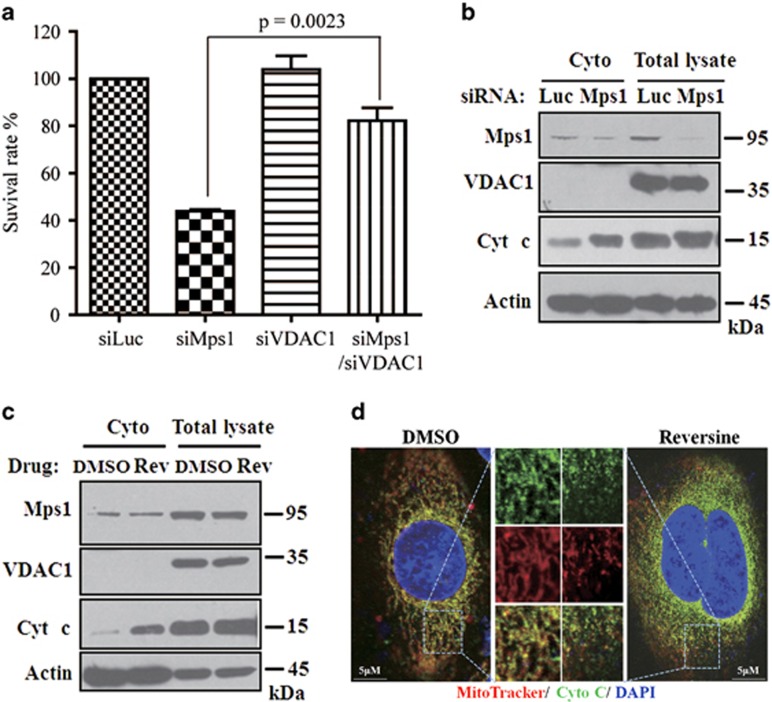
Mps1–VDAC1 interaction regulates cell viability. (**a**) SW480 cells were transfected with siRNA and kept in medium for 4 days. The cell viability was determined by crystal violet staining. The statistical results of the surviving cells are shown and the siRNA efficiency is shown in Supplementary Figure 6e. Data are representative of three independent experiments. Error bars, S.D. (**b** and **c**) SW480 cells were transfected with siRNA (**b**) or treated with Reversine (**c**) and after 4 days the cytoplasmic fraction was separated and the distribution of cytochrome *c* in cytoplasm was detected via western blotting. (**d**) SW480 cells were treated with DMSO or Reversine and then stained with Mitotracker and cytochrome *c* antibody for observing the morphology of mitochondria and the release of cytochrome *c*

Next we set to investigate whether Mps1–VDAC1 interaction regulates cell viability. SW480 cells were treated with VDAC1 siRNA plus Mps1 siRNA or Reversine. VDAC1 depletion can significantly counteract the Mps1 siRNA- or Reversine-induced cell death (Figure 7a and Supplementary Figures 6g and h). VDAC1 modulates the release of cytochrome *c*.^52^ We then tested whether Mps1 participates in the regulation of cytochrome *c* release. As shown, inactivation of Mps1 by either Reversine or Mps1 siRNA can promote the release of cytochrome **c** into the cytoplasm (Figures 7b–d), suggesting that Mps1 activation plays an inhibitory effect on cytochrome *c* release.

Collectively, we provide a mechanism that high levels of Mps1 promote cell survival in aneuploid tumor cells. Mps1 is activated on the kinetochore to assembly MCC, allowing tumor cells to keep a basic level of the SAC to maintain genome instability; meanwhile, another fraction of active Mps1 is recruited to mitochondria by binding to VDAC channels that inhibits the release of cytochrome *c* and eventually prevents aneuploid tumor cells from dying from various stresses.

## Discussion

Numerous studies have shown that SAC is essential for cell viability.^5, 6, 53, 54^ However, in these studies SAC proteins were constitutively disturbed, raising the possibility that other signaling pathways could be affected as several of them have functions beyond SAC regulation.^12, 13, 14^ Though it is not known whether a single SAC disruption affects cell viability, it has been suggested that cell fate depends on the degree of SAC disruption.^55^ In this paper we found that the amount of Mps1 required for cell survival is far more than that of maintaining SAC in aneuploid cell lines, suggesting that besides the SAC feature, other functions of Mps1 also maintain cell viability. Using a short-term Mps1 inhibition strategy, we revealed that single SAC depletion is sufficient to induce cell death that relies on successful subsequent cytokinesis. Deprivation of Mps1 functions in mitosis not only causes SAC depletion but also induces mitochondrial morphological change. Notably, Mps1 can directly regulate the release of cytochrome *c* by binding to mitochondrial protein VDAC1. The C-terminal of Mps1 is essential for SAC signal transduction, VDAC1 association and cell survival. Based on these findings, we postulate that high levels of Mps1 selectively attribute to survival of aneuploid cancer cells by regulating the functions of SAC and mitochondria. This model gives an explanation to why aneuploid tumor cells are addicted to high levels of Mps1.

The coordination between mitotic progression and mitochondria was observed several decades ago.^56^ The morphology of mitochondria is regulated by several cell cycle regulators, including cyclin B1–cyclin-dependent kinase 1 (CDK1) and Aurora A, exerting their function by phosphorylating Drp1 (dystrophin-related protein 1) and RALA (V-ral simian leukemia viral oncogene homolog A).^57, 58^ Our data shows that several SAC regulators, including Mps1, Hec1 and Bub3, can locate to mitochondria. Although VDAC is required for full mitochondrial recruitment of Mps1, we cannot exclude that other molecules are also involved in this process. One of the candidate may be Mortalin that can bind to Mps1.^51^ Hec1 is another candidate that also appears in mitochondrial fractions. We suspect that Hec1 binds and recruits Mps1 to the kinetochore and mitochondria. In fact, we also found that transcriptional levels of several mitochondrial proteins are altered upon inhibition of Mps1 kinase, including SLC25A13 (solute carrier family 25, member 13), NDUFB6 (NADH: ubiquinone oxidoreductase subunit B6), HSD3B1 (hydroxy-*Δ*-5-steroid dehydrogenase, 3 *β*- and steroid *Δ*-isomerase 1), CASP7 (caspase 7), ATP5F1 (ATP synthase, H+ transporting, mitochondrial Fo complex subunit B1), ATP5L (ATP synthase, H+ transporting, mitochondrial Fo complex subunit G) and ACN9 (ACN9 homolog (*S. cerevisiae*)). These findings suggest that there is a connection between mitochondria and kinetochore.

Many studies have shown that VDAC channels are involved in the life and death of cells. In mouse, VDAC molecules are dispensable for mitochondrial functions and survival as knockouts of one or even three forms of VDAC cannot prevent cells from Bax (BCL2-associated X protein) and Bid (BH3 interacting domain death agonist) activation-induced cell death.^59^ In human cancer cells, VDAC channels are required for cell death induced by several agents including select anticancer drugs on the market or in clinical trial, that is Cisplatin, Endostatin and Erastin; all have been proven to associate with either VDAC1 or VDAC2 molecules.^60^ Accumulating evidence suggests that both VDAC1 and VDAC2 are major mediators of apoptosis whereas VDAC3 has functions beyond that, exemplified by the Fisk lab discovery that Mps1 binds VDAC3 to regulate centriole assembly and ciliogenesis.^26, 61^ VDAC1 is a unique structural class of *β*-barrel membrane proteins consisting of 19 transmembrane *β*-strands and a flexible N-terminal fragment.^62^ The N-terminal fragment serves as a binding site platform through which VDAC-binding proteins – such as GSK3 (glycogen synthase kinase 3), hexokinase, GAPDH (glyceraldehyde-3-phosphate dehydrogenase) and Tubulin – can bind and regulate the flux of metabolites and cytochrome *c*.^52^ The Fisk lab recently discovered that VDAC1 can localize to centrosomes.^63^ Our result confirmed their find (Supplementary Figure 6f). In this paper, we identified Mps1 as a novel regulator of VDAC1; Mps1 binding to VDAC1 can attribute to tumor cell survival by regulating cytochrome *c* release. Recently, it was reported that VDAC channels are involved in a newly discovered type of cell death, ferroptosis, upon oxidative stress.^64, 65, 66^ It would be interesting to investigate whether Mps1 is involved in ferroptosis via VDAC channels in the future.

## Materials and Methods

### Constructs and stable cell lines

All ectopic Mps1 expression constructs express a siRNA-insensitive allele and were generated as described previously.^67^ Mps1 mutants, including FTH tagged Mps1ΔD, Cyclin B degradation fragment fused Mps1 and degMps1, were generated via a standard protocol. To build the nondegMps1, the D-box motif responsible for Cyclin B degradation in degMps1 was deleted using a QuikChange mutagenesis kit (Agilent, La Jolla, CA, USA). All Mps1 constructs for stable expression were cloned into pREX-IRES-Hygromycin (Hygro), a derivative of the bicistronic retroviral vector pREX-IRES-GFP described previously.^68^ Stable cell lines in SW480 were generated following the procedure as described previously.^67^ To purify Mps1 protein, the FTH tagged Mps1 was inserted into vector pEXL-FTH and expressed in 293T cells using a transient transfection procedure.

### Cell culture, transfection and drug treatment

SW480, HT29, LoVo and HCT116 cells were purchased from American Type Culture Collection (Manassas, VA, USA) and were maintained following standard protocols. All siRNA duplexes used in this study were purchased from GenePharma (Shanghai, China). The details of the siRNA duplexes are shown in Supplementary Table S2. Transfection of siRNA duplexes was conducted with Transfectin (Bio-Rad, Hercules, CA, USA) according to the manufacturer's protocol and subjected to western blot analysis or subsequent treatments as indicated in the main text.

### Chromosomal spreading

To validate the colon cancer cell lines, the karyotypes of each cell line were examined as previously reported.^35^ Briefly, the mitotic cells, synchronized at prometaphase by 100 ng/ml Nocodazole treatment for 12 h, were collected by the shaking-off method. 2 × 10^5^/ml cells were incubated in a hypotonic buffer (50 mM Tris (pH 7.4) and 55 mM KCl), fixed with freshly made Carnoy's solution (75% methanol and 25% acetic acid), dropped onto glass slides and dried at 80 °C. The metaphase plate was revealed by counterstaining with staining buffer (1.5 *μ*g/ml 4',6-diamidino-2-phenylindole (DAPI) and 50% glycerol in Dulbecco's phosphate-buffered saline (D-PBS)) and then imaged using a confocal microscope. To prepare a representative figure of chromosome spread, the pictures were inversed and the contrast was adjusted using Adobe Photoshop CS2 software.

### Mitochondrial and cytoplasmic fractionation

The mitochondrial fraction was isolated using a dounce homogenization from a kit (89874, Life Technologies, Waltham, MA, USA) following the manufacturer's protocol. Briefly, cells were harvested by trypsin digest, mitochondria isolation reagent A was added followed by incubation on ice for exactly 5 min. Then, the cell suspension was transferred to the Dounce tissue grinder and homogenized on ice. A sufficient number of strokes (50 for SW480 cells) was performed to effectively lyse the cells. The lysed cells were centrifuged at 600 × *g* for 10 min at 4 °C. The supernatant was transferred to a new tube at 12 000 × *g* for 10 min at 4 °C. The supernatant (cytosol fraction) was then transferred to a new tube. The pellet contains the isolated mitochondria, resuspended in mitochondria isolation reagent B.

### Immunoprecipitation assay

SW480 cells stably expressing FTHMps1 were lysed in buffer (20 mM Tris-Cl (pH 8.0), 0.2 M NaCl, 0.5% NP40, 1 mM EDTA, 1 mM PMSF, 20 mM NaF, 0.1 mM Na_3_VO_4_ and 1 × Protease inhibitors (Roche)) for 5 min before scraping. The cell extracts were clarified by spinning at 13 000 r.p.m. for 10 min and incubated with Anti-FLAG affinity gel (Sigma-Aldrich) for 1 h. The beads were collected by centrifugation at 500 × *g* for 1 min and washed once using 1 ml NETN buffer. All of the samples were finally processed by adding 30 *μ*l of 1 × SDS sample buffer with *β*-mercaptoethanol and boiled for 5 min. Proteins in one half of each sample were resolved by SDS-PAGE and subjected to immunoblot analysis.

### Immunoblot, immunofluorescence and immunohistochemistry

Immunoblot and immunofluorescence analyses were performed following standard methods. The antibodies applied for the detection of the proteins of interest include: anti-Mps1 (NT, Millipore, Billerica, MA, USA), anti-VDAC1,2,3 (sc-98708, Santa Cruz, Dallas, TX, USA); anti-VDAC1 (ab154856, Abcam, Cambridge, MA, USA), anti-Cyclin B (SC-245, Santa Cruz); anti-Mad2 (C-9, Santa Cruz), anti-BubR1 (Bethyl, Montgomery, TX, USA), rabbit, anti-Hec1 (9G3, Abcam), Anti-Bub3 (cat. no. 611730, BD Transduction Laboratories, Franklin Lakes, NJ, USA), anti-cytochrome *c* (ab133504, Abcam); anti-Aurora B (ab45145, Abcam), anti-Histone 3 (ab1791, Abcam), anti-pS10 Histone 3(ab32107, Abcam), anti-Aurora B pT232 (Rockland, Limerick, PA, USA) and anti-GAPDH (G-9, Santa Cruz). For immunohistochemistry, the paraffin-embedded colon cancer tissues spotted on a chip (BC05023-54 dots, AlenabioInc, Xi'an, China) were subjected to heat-induced epitope retrieval in Tris–ethylene diaminete traacetic acid buffer (pH 9.0). The slide was incubated in an anti-Mps1 (Millipore, anti-NT) antibody in a dilution of 1 : 100 for 2 h. Detection was performed with a DAB IHC Detection System (Invitrogen, Grand Island, NY, USA). Images were acquired with Leica SCN400 slide scanner (Buffalo Grove, IL, USA) and then arranged in Photoshop software. The quantification of Mps1 levels in cancer tissues and tumor grading was performed manually following standard methods.

### Cell viability assay

The cells were fixed with enough 70% ethanol to cover the cells and incubated for 30 min at room temperature. The ethanol was then removed and incubated with sufficient amount of crystal violet solution (BD, Franklin Lakes, NJ, USA, for microorganism staining) for 1 h at room temperature. The crystal violet was removed and then washed away by running a stream of distilled water over the plate. To generate a representative figure, the plate was filled with 10% glycerol and scanned. To quantify the remaining number of cells, the crystal violet in the plate was dissolved in methanol for 10 min and then analyzed by an ELISA reader.

### Time-lapse fluorescence imaging

SW480 cells stably expressing H2B-GFP (SW480-H2BGFP) were seeded in an eight-chambered cover glass (Lab-Tek Chambered no 1.0 Borosilicate Cover Glass System, Nunc, Grand Island, NY, USA) in Dulbecco's modified Eagle's medium (DMEM) at 50% confluence. SW480-H2BGFP cells were synchronized at prometaphase by a single thymidine arrest along with a Nocodazole treatment procedure. Mitotic cells were collected by with a shaking-off method and released into and maintained in D-PBS. Before imaging, the cells were released into fresh DMEM with chemicals as suggested and transferred to the chamber mounted on the microscope. The images were taken at 5 min intervals and the representative images of the slices were prepared using Image J software (Bethesda, MD, USA).

### Flow cytometry assay

For DNA content determination, SW480 cells treated as indicated in the figure legend were trypsinized, washed three times with D-PBS, fixed with 70% ethanol and stored at −20 °C. Fixed cells were then resuspended in D-PBS containing 10 mg/ml RNase A and 20 mg/ml PI for 30 min at 37 °C before flow cytometry. For the quantification of endogenous reactive oxygen species (ROS) levels, cells were treated with chemicals as indicated and then incubated with 10 *μ*M H2DCF for 10 min before the samples were collected at the indicated time points. The cells were then subjected to flow cytometric analysis using a FACSCalibur (Becton Dickinson, Tokyo, Japan) with the corresponding channels following a standard method.

### Microarray analysis and quantitative PCR validation

Total RNA was isolated from the treated cells using TRIzol reagent (Life Technologies) following the manufacturer's instructions. The concentration and quality of the isolated RNA was initially assessed with a NanoDrop ND-1000 spectrophotometer (Thermo Fisher Scientific, Grand Island, NY, USA). Total RNA samples were sent out to OE Biotech Company (Shanghai, China) and then applied to Affymetrix Human HTA2.0 GeneChip (Agilent Technologies, Santa Clara, CA, USA) to assess the mRNA expression profiles. Raw data were preprocessed using Affymetrix GeneChip Command Console Software (version 4.0, Affymetrix). Next, GO terms enriched KEGG pathways were calculated. A heat map of differential expressed genes was generated using MeV.4.9 software (La Jolla, CA, USA). Quantitative reverse transcription-PCR (qRT-PCR) of the selected genes was completed. The total RNA used for qRT-PCR was isolated from the same samples used for the microarray, and the first-strand cDNA was synthesized for each of the selected genes with SuperScript III Reverse Transcriptase (Life Technologies) according to the manufacturer's instructions. The gene expression levels were quantified using 2 × SYBR Green PCR Master mix (ABI, Grand Island, NY, USA) on an iQ5 Real-Time PCR Detection System (Bio-Rad). Primers for qRT-PCR are listed in Supplementary Table 3.

## Abbreviations

ACN9: ACN9 homolog (*S. cerevisiae*)
APC/C: anaphase-promoting complex/cyclosome
ATG8: autophagy-related 8
ATP5F1: ATP synthase, H+ transporting, mitochondrial Fo complex subunit B1
ATP5L: ATP synthase, H+ transporting, mitochondrial Fo complex subunit G
Bax: BCL2-associated X protein
Bid: BH3 interacting domain death agonist
Bub1/3: budding uninhibited by benzimidazoles 1/3 homolog
BubR1: Bub1-related kinase
CASP7: caspase 7
CENP-E: centromere protein E
DAPI: 4',6-diamidino-2-phenylindole
CDC20: cell division cycle 20
CDK1: cyclin-dependent kinase 1
DMEM: Dulbecco's modified Eagle's medium
CPC: chromosomal passenger complex
D-PBS: Dulbecco's phosphate-buffered saline
Drp1: dystrophin-related protein 1
GAPDH: glyceraldehyde-3-phosphate dehydrogenase
GSK3: glycogen synthase kinase 3
GZMB: granzyme B
Hec1: basic helix-loop-helix (bHLH) DNA-binding superfamily protein HECATE 1
HSD3B1: hydroxy-*Δ*-5-steroid dehydrogenase, 3 *β*- and steroid *Δ*-isomerase 1
Knl1: kinetochore-null protein 1
Mad1/2: mitotic arrest deficient-like 1/2
MCC: mitotic checkpoint complex
Mps1: monopolar spindle 1
NDUFB6: NADH: ubiquinone oxidoreductase subunit B6
Plk1: polo-like kinase 1
qRT-PCR: quantitative reverse transcription-PCR
RALA: V-ral simian leukemia viral oncogene homolog A
ROS: reactive oxygen species
SAC: spindle assembly checkpoint
SLC25A13: solute carrier family 25, member 13
VDAC1: voltage-dependent anion channel 1
