# Supplementary material for: Mps1 kinase regulates tumor cell viability via its novel role in mitochondria
Source: Cell Death Dis. 2016 Jul 7;7(7):e2292–. doi: 10.1038/cddis.2016.193 (PMC4973343; doi:10.1038/cddis.2016.193)
Supplement: Supplementary Information [file cddis2016193x1.docx]

**Supplementary Information**

**­­­­Mps1 kinase regulates tumor cell viability via its novel role in mitochondria**

Xiaojuan Zhang^1, 5^, Youguo Ling^2, 5^, Yu Guo^1, 4^, Yuanyuan Bai^2^, Xiaoqian Shi^1, 4^, Fuxing Gong^1, 4^, Pingping Tan^1^, Yanhong Zhang^2^, Congwen Wei^2^, Xiang He^2^, Adrian Ramirez^3^, Xuedong Liu^3^, Cheng Cao^2^, Hui Zhong^2, 6^, Quanbin Xu^2, 6^* , Runlin Z Ma^1, 4, 6^*

^1^State Key Laboratory for Molecular Developmental Biology, Institute of Genetics and Developmental Biology, Chinese Academy of Sciences, Beijing 100101, China; ^2^Beijing Institute of Biotechnology, Beijing 100850, China; ^3^University of Colorado at Boulder, Boulder 80302, Colorado, USA; ^4^Graduate school, University of the Chinese Academy of Sciences, Beijing 100149, China. ^5^These authors contributed equally to this work. ^6^These authors contributed equally to this work.

*Corresponding authors: **Runlin Z Ma,** Institute of Genetics and Developmental Biology, Chinese Academy of Sciences, Beijing 100101, China, E-mail: rlma@genetics.ac.cn

**Quanbin Xu**, Beijing Institute of Biotechnology, Beijing 100850, China; E-mail: [xuquanbin73@yahoo.com](mailto:xuquanbin73@yahoo.com).

**Running title:** High levels of Mps1 and cancer cell survival.

**Supplementary Figure**

**
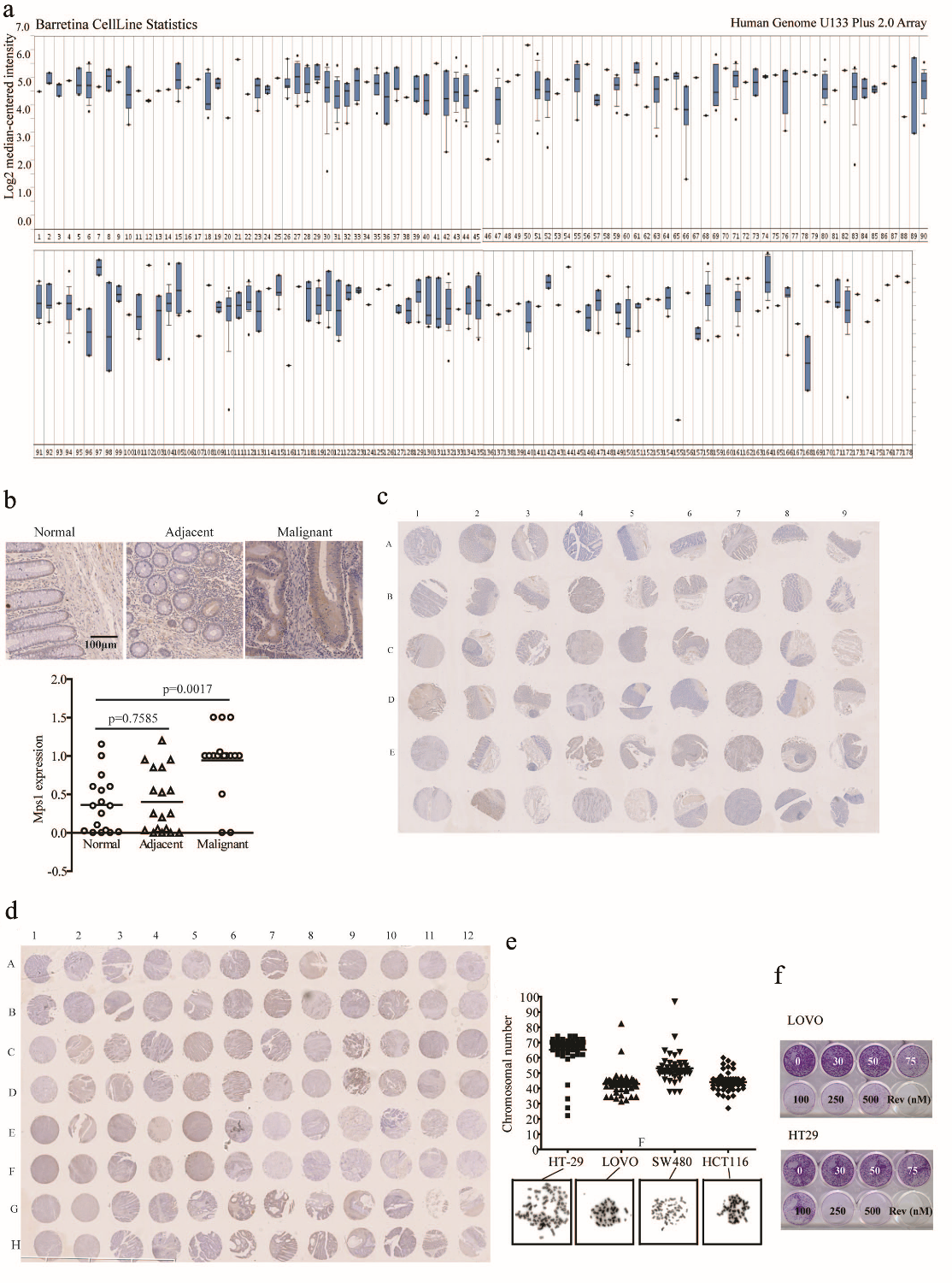
**

**Supplementary Figure 1** Mps1 is overexpressed in a variety of tumors. **(a)** The mRNA level of Mps1 in a set of tumors. The data were retrieved from an annotated microarray data set and the figure was generated using Oncomine version 4.0 (<https://www.oncomine.com/resource/login.html>). The labels for tumor tissues are as follows: 1. Acute Biphenotypic Leukemia (1)；2. Acute Lymphoblastic Leukemia (3)；3. Acute Megakaryoblastic Leukemia (3)；4. Acute Monoblastic Leukemia (1)；5. Acute Monocytic Leukemia (6)；6. Acute Myeloid Leukemia (15)；7. Acute Myelomonocytic Leukemia (1)；8. Acute Promyelocytic Leukemia (3)；9. Acute Undifferentiated Leukemia (1)；10. Adenosquamous Lung Carcinoma (4)；11. Adrenal Gland Neuroblastoma (1)；12. Amelanotic Skin Melanoma (2)；13. Ampulla of Vater Adenocarcinoma (1)；14. Anaplastic Astrocytoma (1)；15. Anaplastic Large Cell Lymphoma (6)；16. Anaplastic Oligodendroglioma (1)；17. B Lymphoblastic Lymphoma (1)；18. B-Cell Acute Lymphoblastic Leukemia (10)；19. B-Cell Non-Hodgkin's Lymphoma (2)；20. B-Cell Prolymphocytic Leukemia (1)；21. Barrett's Adenocarcinoma (1)；22. Bile Duct Carcinoma (1)；23. Bladder Cancer (7)；24. Bladder Papillary Urothelial Carcinoma (2)；25. Bladder Squamous Cell Carcinoma (1)；26. Bladder Urothelial Carcinoma (10)；27. Blast Phase Chronic Myelogenous Leukemia (13)；28. Bone Osteosarcoma (9)；29. Brain Astrocytoma (6)；30. Brain Glioblastoma (19)；31. Breast Adenocarcinoma (18)；32. Breast Carcinoma (6)；33. Bronchioloalveolar Carcinoma (7)；34. Bronchogenic Carcinoma (1)；35. Burkitt's Lymphoma (10)；36. Cecum Adenocarcinoma (3)；37. Cecum Carcinoma (5)；38. Cerebral Glioblastoma (1)；39. Chondrosarcoma (2)；40. Chronic Lymphocytic Leukemia (3)；41. Chronic Myelogenous Leukemia (1)；42. Clear Cell Renal Cell Carcinoma (6)；43. Colon Adenocarcinoma (27)；44. Colon Carcinoma (14)；45. Colon Mucinous Adenocarcinoma (1)；46. Conventional Chondrosarcoma (1)；47. Cutaneous Melanoma (30)；48. Cutaneous T-Cell Non-Hodgkin's Lymphoma (1)；49. Dedifferentiated Chondrosarcoma (1)；50. Desmoplastic Medulloblastoma (1)；51. Diffuse Large B-Cell Lymphoma (15)；52. Ductal Breast Carcinoma (10)；53. Duodenal Adenocarcinoma (1)；54. Embryonal Rhabdomyosarcoma (1)；55. Endometrial Adenocarcinoma (9)；56. Endometrial Adenosquamous Carcinoma (1)；57. Endometrial Carcinoma (2)；58. Endometrial Clear Cell Adenocarcinoma (1)；59. Endometrial Endometrioid Adenocarcinoma (10)；60. Epithelioid Mesothelioma (1)；61. Erythroleukemia (4)；62. Esophageal Adenocarcinoma (1)；63. Esophageal Squamous Cell Carcinoma (23)；64. Essential Thrombocythemia (1)；65. Ewing's Sarcoma (4)；66. Ewing's Sarcoma of Bone (4)；67. Fibrosarcoma (1)；68. Gallbladder Carcinoma (1)；69. Gastric Adenocarcinoma (11)；70. Gastric Adenosquamous Carcinoma (1)；71. Gastric Cancer (16)；72. Gastric Diffuse Large B-Cell Lymphoma (1)；73. Gastric Small Cell Neuroendocrine Carcinoma (2)；74. Gastric Tubular Adenocarcinoma (2)；75. Gingival Squamous Cell Carcinoma (1)；76. Glioblastoma (7)；77. Gliosarcoma (1)；78. Head and Neck Basaloid Carcinoma (1)；79. Hepatoblastoma (1)；80. Hepatocellular Carcinoma (23)；81. Hilar Cholangiocarcinoma (1)；82. Histiocytoma (1)；83. Hodgkin's Lymphoma (13)；84. Hypopharyngeal Squamous Cell Carcinoma (2)；85. Immunoblastic Lymphoma (2)；86. Infiltrating Bladder Urothelial Carcinoma (1)；87. Infiltrating Renal Pelvis Urothelial Carcinoma, Sarcomatoid Variant (1)；88. Intracranial Melanoma (1)；89. Intrahepatic Cholangiocarcinoma (3)；90. Invasive Ductal Breast Carcinoma (18)；91. Large Cell Lung Carcinoma (8)；92. Laryngeal Squamous Cell Carcinoma (4)；93. Liver Alveolar Rhabdomyosarcoma (1)；94. Lung Adenocarcinoma (57)；95. Lung Carcinoid Tumor (1)；96. Lung Cancer (2)；97. Lung Giant Cell Carcinoma (2)；98. Malignant Giant Cell Tumor of Bone (3)；99. Malignant Glioma, NOS (3)；100. Malignant Ovarian Brenner Tumor (1)；101. Mantle Cell Lymphoma (5)；103. Medulloblastoma (3)；104. Melanoma (24)；105. Mesothelioma (6)；106. Metaplastic Breast Carcinoma (1)；107. Mixed Glioma (1)；108. Mixed Mesodermal (Mullerian) Tumor (1)；109. Mixed Small Cell and Squamous Cell Lung Carcinoma (2)；110. Multiple Myeloma (21)；111. Mycosis Fungoides (2)；112. Neuroblastoma (16)；113. Neuroglial Tumor, NOS (2)；114. Non-Hodgkin's Lymphoma (1)；115. Non-Small Cell Lung Carcinoma (9)；116. Oligoastrocytoma (1)；117. Oral Cavity Mucoepidermoid Carcinoma (1)；118. Oral Cavity Squamous Cell Carcinoma (3)；119. Ovarian Adenocarcinoma (8)；120. Ovarian Carcinoma (5)；121. Ovarian Clear Cell Adenocarcinoma (6)；122. Ovarian Cystadenocarcinoma (2)；123. Ovarian Endometrioid Adenocarcinoma (2)；124. Ovarian Granulosa Cell Tumor (1)；125. Ovarian Leiomyosarcoma (1)；126. Ovarian Mixed Epithelial Tumor (1)；127. Ovarian Mucinous Adenocarcinoma (3)；128. Ovarian Mucinous Cystadenocarcinoma (2)；129. Ovarian Serous Adenocarcinoma (4)；130. Ovarian Serous Cystadenocarcinoma (5)；131. Ovarian Serous Surface Papillary Carcinoma (3)；132. Pancreatic Adenocarcinoma (21)；133. Pancreatic Adenosquamous Carcinoma (1)；134. Pancreatic Carcinoma (8)；135. Pancreatic Ductal Adenocarcinoma (12)；136. Pancreatic Endocrine Carcinoma (1)；137. Papillary Lung Adenocarcinoma (1)；138. Papilloma (1)；139. Pharyngeal Carcinoma (1)；140. Plasma Cell Leukemia (4)；141. Plasmacytoma (1)；142. Pleomorphic Hepatocellular Carcinoma (2)；143. Pleural Biphasic Mesothelioma (1)；144. Pleural Mesothelioma (1)；145. Primary Effusion Lymphoma (1)；146. Prostate Adenocarcinoma (2)；147. Prostate Carcinoma (4)；148. Prostate Small Cell Carcinoma (1)；149. Rectal Adenocarcinoma (6)；150. Renal Carcinoma (8)；151. Renal Cell Carcinoma (5)；152. Renal Leiomyoma (1)；153. Rhabdoid Tumor of the Kidney (1)；154. Rhabdomyosarcoma (7)；155. Scirrhous Breast Carcinoma (1)；156. Sezary Syndrome (1)；157. Signet Ring Cell Gastric Adenocarcinoma (2)；158. Small Cell Lung Carcinoma (49)；159. Small Lymphocytic Lymphoma (1)；160. Squamous Cell Breast Carcinoma, Acantholytic Variant (1)；161. Squamous Cell Lung Carcinoma (23)；162. Submandibular Gland Squamous Cell Carcinoma (1)；163. Supraglottic Squamous Cell Carcinoma (1)；164. T-Cell Acute Lymphoblastic Leukemia (15)；165. T-Cell Large Granular Lymphocyte Leukemia (1)；166. Thyroid Gland Follicular Carcinoma (4)；167. Thyroid Gland Medullary Carcinoma (1)；168. Thyroid Gland Papillary Carcinoma (2)；169. Thyroid Gland Sarcoma (1)；170. Thyroid Gland Squamous Cell Carcinoma (1)；171. Thyroid Gland Undifferentiated (Anaplastic) Carcinoma (3)；172. Tongue Squamous Cell Carcinoma (15)；173. Undifferentiated Gastric Carcinoma (1)；174. Undifferentiated Ovarian Carcinoma (1)；175. Ureter Urothelial Carcinoma (1)；176. Uterine Corpus Endometrial Stromal Sarcoma (1)；177. Uterine Corpus Leiomyosarcoma (1)；178. Vulvar Leiomyosarcoma. **(b and c)** The representative figures of the matched normal, adjacent and colorectal cancer tissues stained with an antibody against Mps1. the statistical result of the quantification of Mps1 levels from 18 subjects. The original image of the tumor tissue array is shown in **c**. The scores of immunohistochemical staining are listed in table S4. **(d)** The original image of the tumor tissue array whose representative figures and statistical result are listed in figure 1a. The slides were treated following a standard protocol and stained with an anti-Mps1 antibody (N1, Abcam) at a dilution of 1:100. **(e)** The karyotypes of four colon cancer cell lines. The chromosome spread of four colon cancer cell lines was prepared and quantified in a standard method. **(f)** Two colorectal cancer cell lines, LoVo and HT29, were treated with the Mps1 inhibitor Reversine at escalating doses and the cell viability were determined via crystal violet staining. The result of cell viability was present in Figure 1d.

**
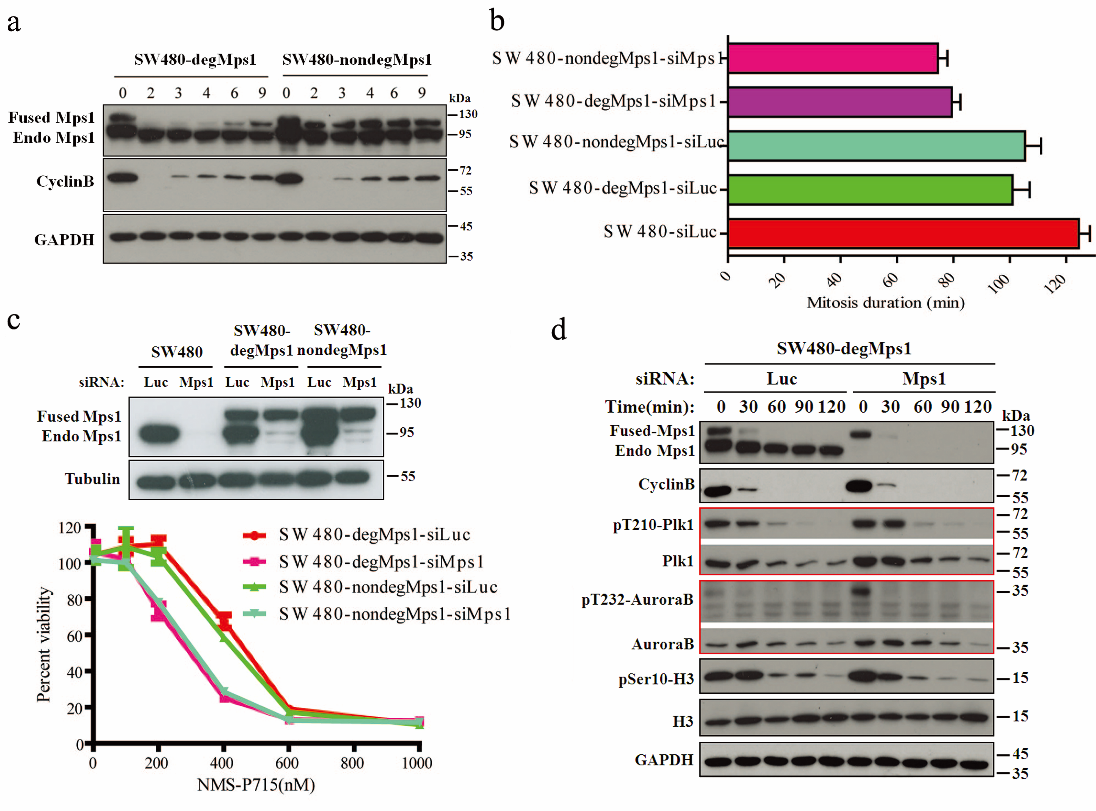
**

**Supplementary Figure 2** High levels of Mps1 promote tumor cells survival irrelevant to its role in cytokinesis. **(a)** SW480-degMps1 and SW480-undegMps1 were released from Nocodazole and subjected to western blotting with the indicted antibodies at the indicated time points. **(b)** SW480-degMps1 and SW480-undegMps1 were transfected with Mps1 siRNA for 48 hours and the cells were then synchronized at the late G2 phase before the mitotic progression is chased by live cell imaging. Data are representative of three independent experiments. Error bars, s.d. **(c)** Equal numbers of SW480-degMps1 and SW480-undegMps1 cells were transfected twice with Mps1 siRNA and the cells were then released into medium with NMS-P715 after 48 hours; the remaining cells were revealed by crystal violet staining after another 5 days. The upper panel shows the efficiency of siRNA knockdown and the lower panel shows the percentage of surviving cells. Data are representative of three independent experiments. Error bars, s.d. **(d)** Cells SW480-degMps1 and SW480-undegMps were transfected with siRNA as shown and the cells were arrested at prometaphase and then released into fresh medium. The cells were then collected at the indicated time points and subjected to western blot analysis with indicated antibodies.

**
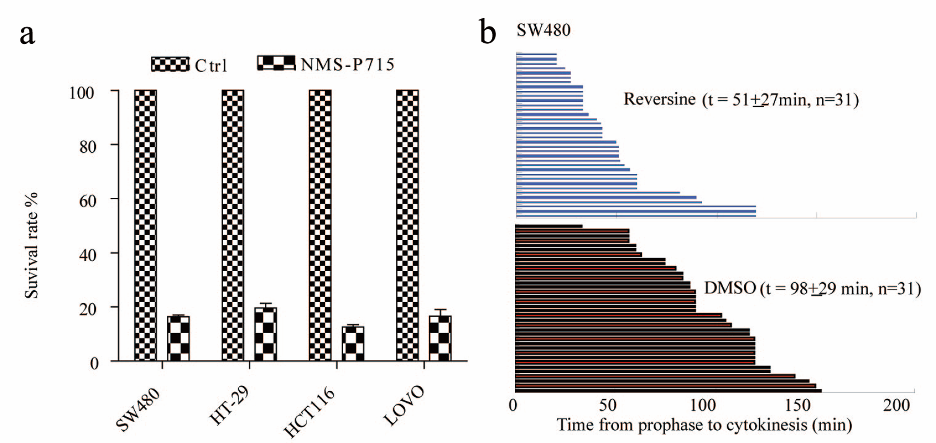
**

**Supplementary Figure 3** Single loss of SAC by temporary Mps1 inhibition is sufficient to cause cell death. **(a)** Four colorectal cancer cell lines, including HCT116, LoVo, HT29 and SW480, were treated with the Mps1 inhibitor NMS-P715 at escalating doses for 24 hours and the cell viability were determined via crystal violet staining. Data are representative of three independent experiments. Error bars, s.d. **(b)** SW480 cells were treated with Reversine or DMSO for two hours and the time span from prophase to cytokinesis was recorded.

**
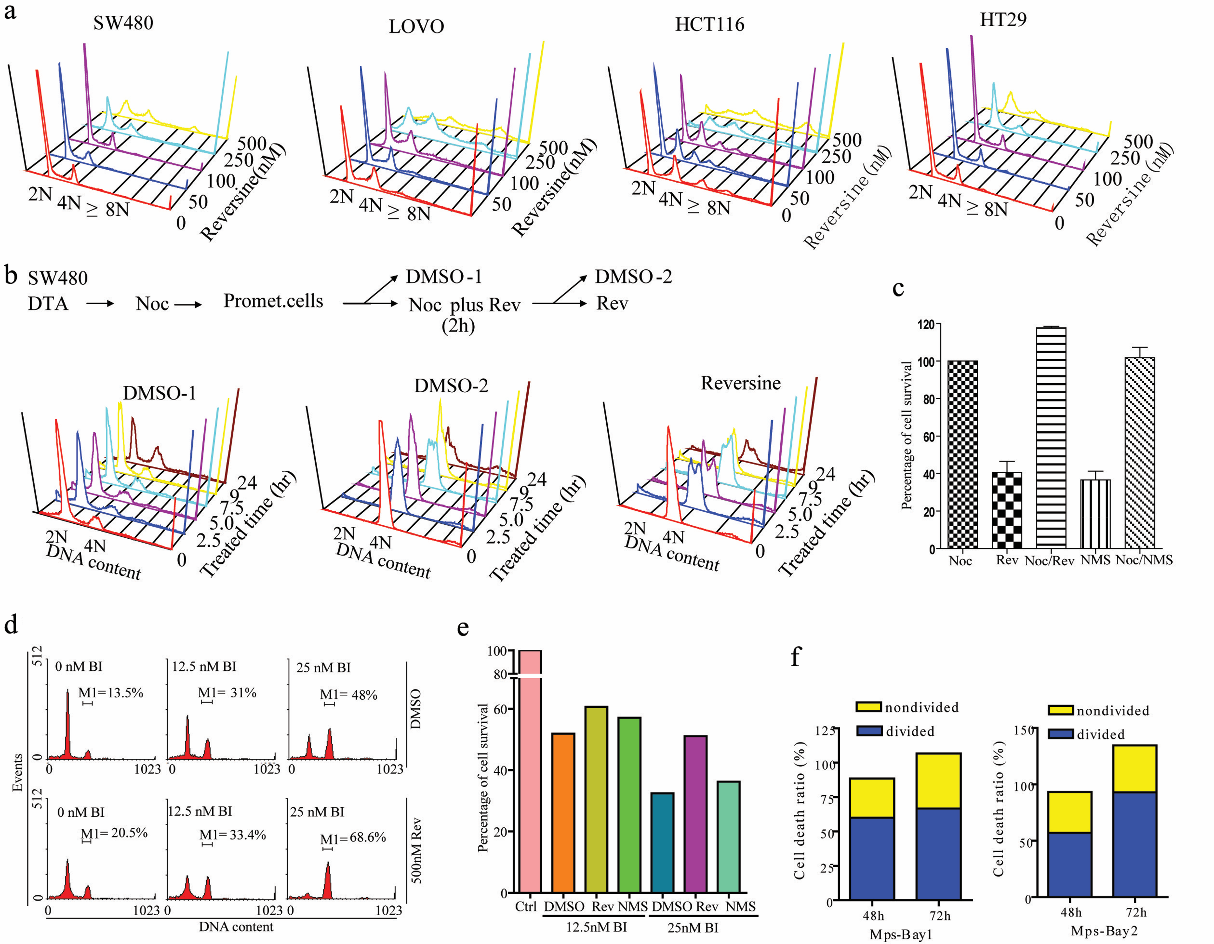
**

**Supplementary Figure 4** Loss of SAC by Mps1 inhibition prefers to killing tumor cells undergoing successful cytokinesis. **(a)** Four colorectal cancer cell lines, including HCT116, LoVo, HT29 and SW480, were treated with the Mps1 inhibitor Reversine at escalating doses and the DNA content was measured by flow cytometry. **(b)** SW480 cells were treated following the shown procedure and then the DNA content was measured by flow cytometry. **(c)** SW480 cells were synchronized at prometaphase in Nocodazole for 12 hours and treated with chemicals as indicated for 2 hours prior to release into fresh medium for 6 days. The cell viability was determined by crystal violet staining and the quantitative results were generated with Graphpad. This experiment was repeated for three times. Error bars, s.d. **(d and e)** SW480 cells were co-treated with BI 2536 and Reversine and then DNA content and cell viability was measured by flow cytometry (d) and crystal violet staining (e). **(f)** The published data[^42^](#_ENREF_35) in which the fate of HCT116 cells was traced by live cell imaging in the presence of other Mps1 inhibitors were analyzed and shown that SW480 cell death ratio of the diploid cells is much higher than those tetraploid cells in the presence of Mps1 inhibitors.

**
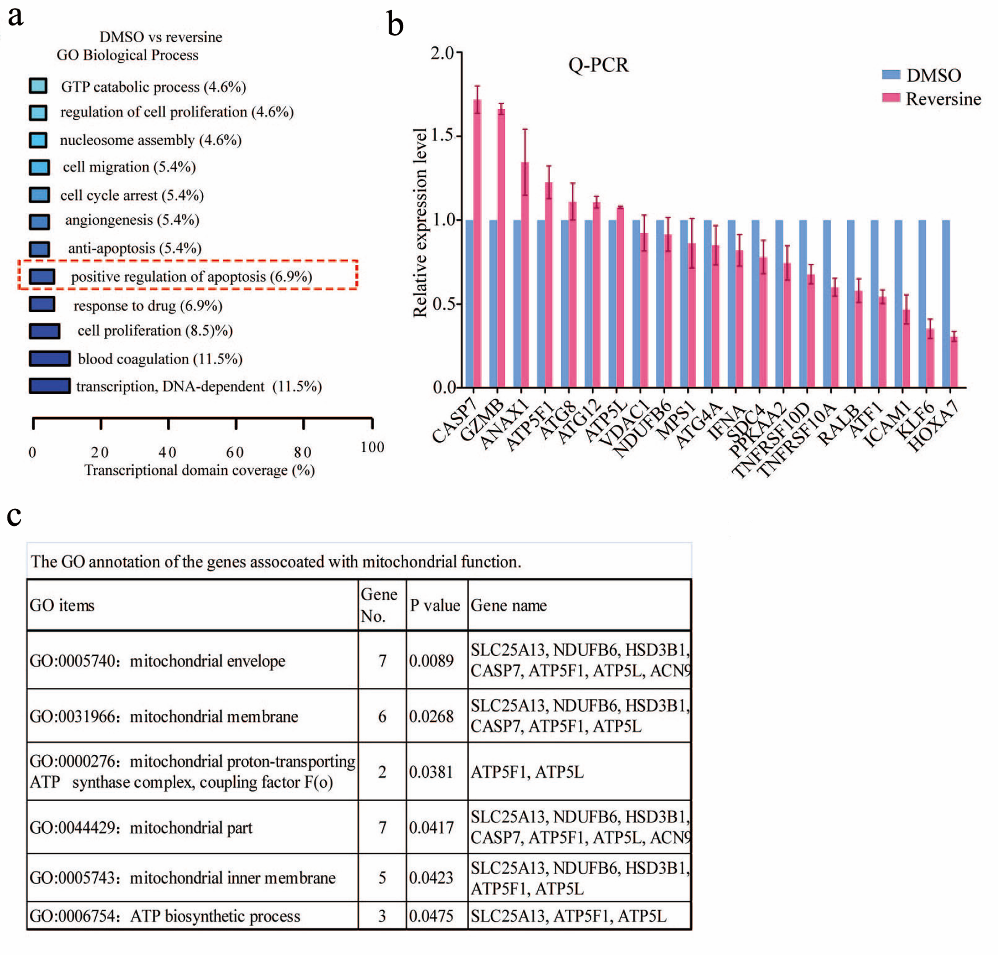
**

**Supplementary Figure 5** Decreased Mps1 levels in tumor cells triggers apoptosis signaling pathway. **(a)** GO analysis of affected gene upon perturbation of Mps1 functions via siRNA. **(b)** SW480 cells were treated with Mps1 inhibitor Reversine and the cells were subjected to RNA extraction and quantitative PCR analysis with the primers against genes as indicated. This experiment was repeated for three times. Error bars, s.d. **(c)** The GO annotation of the genes associated with mitochondrial functions.

**Supplementary Figure 6** Mps1 regulates cell viability by coupling SAC to the mitochondrial functions. **(a)** FTHMps1 and FTHMps1ΔD were overexpressed in 293T cells and after 48 hours cells were lysed. The total cell lysate were subjected to co-immunoprecipitation analysis using Anti-Flag M2 affinity gel and then resolved by Western Blotting and silver staining. A differential band was found and cut to analyze by Mass Spectrometry whose result is shown in table S1. **(b)** FTHMps1 and FTHMps1ΔD were overexpressed in SW480 cells. After 48 hours mitochondria and cytoplasm of SW480 were isolated and then subjected to western blot analysis with indicated antibodies. 20ug of total protein per lane. The distribution ratio of Mito/Cyto of Mps1 was measured in F. Results are expressed as mean ± SEM; ***P* < 0.001. **(c)** SW480 cells treated using indicated siRNA were subjected to immunostaining with antibodies against Mps1 and VDAC1. **(d)** Mitochondria was isolated from SW480 cells and then treated with proteinase K. **(e)** Colocalization of Mps1 and Mortalin. SW480 cells were fixed and stained with antibodies against Mps1 and Mortalin. DNA was counterstained with DAPI. **(f)** SW480 cells were subjected to immunostaining with antibodies against γ-Tubulin and VDAC1. **(g)** SW480 cells were transfected with siRNA as the indicated and kept in medium for 4 days. The cell viability was determined by crystal violates staining. Western blot was used for testing the efficiency of siRNA knockdown. (h) SW480 cells were transfected with VDAC1 siRNA; 24hours later these cells were synchronized at mitosis by Nocodazole treatment for 12 hours; the mitotic cells were collected by shaking-off and washed with PBS three times before reseeded into a 12 wells plate. The remained living cells were quantified with crystal violet staining after 7days.

| **Supplementary Table1** The result of the differential band by Mass Spectrometry. | | | | | | | | |
| --- | --- | --- | --- | --- | --- | --- | --- | --- |
| **No.** | **Scan(s)** | | | **Peptide** | | | | |
| 1 | gi\|23308722\|ref\|NP_003309.2\| dual specificity protein kinase TTK isoform 1 [Homo sapiens] | | | | | | | |
|  | 5738 | | | R.ELTIDSIMNK.V | | | | |
|  | 4782 | | | R.FAELKAIQEPDDAR.D | | | | |
|  | 7423 | | | K.FAFVHISFAQFELSQGNVK.K | | | | |
|  | 7110 | | | K.FAFVHISFAQFELSQGNVKK.S | | | | |
|  | 5238 | | | K.FKNEDLTDELSLNK.I | | | | |
|  | 5337 | | | R.FLYGENM*PPQDAEIGYR.N | | | | |
|  | 5880 | | | R.FLYGENMPPQDAEIGYR.N | | | | |
|  | 8263 | | | R.GAVPLEMLEIALR.N | | | | |
|  | 5814 | | | K.LIDFGIANQM*QPDTTSVVK.D | | | | |
|  | 6279 | | | K.LIDFGIANQMQPDTTSVVK.D | | | | |
|  | 6391 | | | K.NLSASTVLTAQESFSGSLGHLQNR.N | | | | |
|  | 7098 | | | K.NSVPLSDALLNKLIGR.Y | | | | |
|  | 5332 | | | K.YVNLEEADNQTLDSYR.N | | | | |
| 2 | gi\|262399361\|ref\|NP_001160163.1\| dual specificity protein kinase TTK isoform 2 [Homo sapiens] | | | | | | | |
|  | 5738 | | | R.ELTIDSIMNK.V | | | | |
|  | 4782 | | | R.FAELKAIQEPDDAR.D | | | | |
|  | 7423 | | | K.FAFVHISFAQFELSQGNVK.K | | | | |
|  | 7110 | | | K.FAFVHISFAQFELSQGNVKK.S | | | | |
|  | 5238 | | | K.FKNEDLTDELSLNK.I | | | | |
|  | 5337 | | | R.FLYGENM*PPQDAEIGYR.N | | | | |
|  | 5880 | | | R.FLYGENMPPQDAEIGYR.N | | | | |
|  | 8263 | | | R.GAVPLEMLEIALR.N | | | | |
|  | 5814 | | | K.LIDFGIANQM*QPDTTSVVK.D | | | | |
|  | 6279 | | | K.LIDFGIANQMQPDTTSVVK.D | | | | |
|  | 6391 | | | K.NLSASTVLTAQESFSGSLGHLQNR.N | | | | |
|  | 7098 | | | K.NSVPLSDALLNKLIGR.Y | | | | |
|  | 5332 | | | K.YVNLEEADNQTLDSYR.N | | | | |
| **3** | **gi\|4507879\|ref\|NP_003365.1\| voltage-dependent anion-selective channel protein 1 [Homo sapiens]** | | | | | | | |
|  | **6337** | | | **R.EHINLGCDMDFDIAGPSIR.G** | | | | |
|  | **5782** | | | **R.EHINLGCDM*DFDIAGPSIR.G** | | | | |
|  | **7950** | | | **R.GALVLGYEGWLAGYQMNFETAK.S** | | | | |
|  | **5617** | | | **R.GLKLTFDSSFSPNTGK.K** | | | | |
|  | **5294** | | | **R.GLKLTFDSSFSPNTGKK.N** | | | | |
|  | **6750** | | | **K.GYGFGLIKLDLK.T** | | | | |
|  | **5653** | | | **K.LETAVNLAWTAGNSNTR.F** | | | | |
|  | **6465** | | | **K.LTLSALLDGK.N** | | | | |
|  | **5688** | | | **K.LTLSALLDGKNVNAGGHK.L** | | | | |
|  | **5575** | | | **K.TDEFQLHTNVNDGTEFGGSIYQK.V** | | | | |
|  | **6213** | | | **K.WNTDNTLGTEITVEDQLAR.G** | | | | |
|  | **5868** | | | **R.WTEYGLTFTEK.W** | | | | |
|  | **5028** | | | **K.YQIDPDACFSAK.V** | | | | |
| 4 | gi\|14591909\|ref\|NP_000960.2\| 60S ribosomal protein L5 [Homo sapiens] | | | | | | | |
|  | 5791 | | | R.IEGDMIVCAAYAHELPK.Y | | | | |
|  | 5694 | | | K.VFGALKGAVDGGLSIPHSTK.R | | | | |
|  | 7008 | | | K.VGLTNYAAAYCTGLLLAR.R | | | | |
|  | 5400 | | | R.VTNRDIICQIAYAR.I | | | | |
| 5 | gi\|47458811\|ref\|NP_998814.1\| sideroflexin-4 [Homo sapiens] | | | | | | | |
|  | 4617 | | | K.GIAVMDKEGNVLGHSR.I | | | | |
|  | 5208 | | | K.IQSPTEETEIFYHR.G | | | | |
|  | 4489 | | | R.QLLCTNEDVSSPASADQR.I | | | | |
| 6 | gi\|4506723\|ref\|NP_000997.1\| 40S ribosomal protein S3a [Homo sapiens] | | | | | | | |
|  | 5892 | | | K.ACQSIYPLHDVFVR.K | | | | |
|  | 7196 | | | R.VFEVSLADLQNDEVAFR.K | | | | |
|  | 6698 | | | R.VFEVSLADLQNDEVAFRK.F | | | | |
| 7 | gi\|15718687\|ref\|NP_000996.2\| 40S ribosomal protein S3 [Homo sapiens] | | | | | | | |
|  | 6180 | | | R.FGFPEGSVELYAEK.V | | | | |
|  | 4758 | | | R.FIMESGAKGCEVVVSGK.L | | | | |
|  | 5517 | | | R.TEIIILATR.T | | | | |
| 8 | gi\|24234686\|ref\|NP_694881.1\| heat shock cognate 71 kDa protein isoform 2 [Homo sapiens] | | | | | | | |
|  | 5798 | | | K.DAGTIAGLNVLR.I | | | | |
|  | 6032 | | | R.IINEPTAAAIAYGLDK.G | | | | |
|  | 6416 | | | K.SFYPEEVSSMVLTK.M | | | | |
| 9 | gi\|5729877\|ref\|NP_006588.1\| heat shock cognate 71 kDa protein isoform 1 [Homo sapiens] | | | | | | | |
|  | 5798 | | | K.DAGTIAGLNVLR.I | | | | |
|  | 6032 | | | R.IINEPTAAAIAYGLDK.G | | | | |
|  | 6416 | | | K.SFYPEEVSSMVLTK.M | | | | |
| 10 | gi\|16445419\|ref\|NP_005689.2\| secretory carrier-associated membrane protein 3 isoform 1 [Homo sapiens] | | | | | | | |
|  | 5650 | | | K.AQQEFAAGVFSNPAVR.T | | | | |
|  | 4894 | | | R.TAAANAAAGAAENAFR.A | | | | |
| 11 | gi\|16445421\|ref\|NP_443069.1\| secretory carrier-associated membrane protein 3 isoform 2 [Homo sapiens] | | | | | | | |
|  | 5650 | | | K.AQQEFAAGVFSNPAVR.T | | | | |
|  | 4894 | | | R.TAAANAAAGAAENAFR.A | | | | |
| 12 | gi\|13129110\|ref\|NP_077007.1\| methylosome protein 50 [Homo sapiens] | | | | | | | |
|  | 6888 | | | K.VWDLAQQVVLSSYR.A | | | | |
|  | 5544 | | | R.YRSDGALLLGASSLSGR.C | | | | |
| 13 | gi\|16933546\|ref\|NP_444505.1\| 60S acidic ribosomal protein P0 [Homo sapiens] | | | | | | | |
|  | 6902 | | | K.IIQLLDDYPKCFIVGADNVGSK.Q | | | | |
|  | 6950 | | | R.VLALSVETDYTFPLAEK.V | | | | |
| 14 | gi\|4506667\|ref\|NP_000993.1\| 60S acidic ribosomal protein P0 [Homo sapiens] | | | | | | | |
|  | 6902 | | | K.IIQLLDDYPKCFIVGADNVGSK.Q | | | | |
|  | 6950 | | | R.VLALSVETDYTFPLAEK.V | | | | |
| **15** | **gi\|296317337\|ref\|NP_001171712.1\| voltage-dependent anion-selective channel protein 2 isoform 1 [Homo sapiens]** | | | | | | | |
|  | **5425** | | | **K.LTFDTTFSPNTGK.K** | | | | |
|  | **5816** | | | **K.LTLSALVDGK.S** | | | | |
| **16** | **gi\|296317339\|ref\|NP_001171752.1\| voltage-dependent anion-selective channel protein 2 isoform 2 [Homo sapiens]** | | | | | | | |
|  | **5425** | | | **K.LTFDTTFSPNTGK.K** | | | | |
|  | **5816** | | | **K.LTLSALVDGK.S** | | | | |
| **17** | **gi\|42476281\|ref\|NP_003366.2\| voltage-dependent anion-selective channel protein 2 isoform 2 [Homo sapiens]** | | | | | | | |
|  | **5425** | | | **K.LTFDTTFSPNTGK.K** | | | | |
|  | **5816** | | | **K.LTLSALVDGK.S** | | | | |
| 18 | gi\|4507729\|ref\|NP_001060.1\| tubulin beta-2A chain [Homo sapiens] | | | | | | | |
|  | 6181 | | | R.AILVDLEPGTMDSVR.S | | | | |
|  | 6014 | | | K.LAVNMVPFPR.L | | | | |
| 19 | gi\|29788785\|ref\|NP_821133.1\| tubulin beta chain [Homo sapiens] | | | | | | | |
|  | 6181 | | | R.AILVDLEPGTMDSVR.S | | | | |
|  | 6014 | | | K.LAVNMVPFPR.L | | | | |
| 20 | gi\|29788768\|ref\|NP_821080.1\| tubulin beta-2B chain [Homo sapiens] | | | | | | | |
|  | 6181 | | | R.AILVDLEPGTMDSVR.S | | | | |
|  | 6014 | | | K.LAVNMVPFPR.L | | | | |
| 21 | gi\|50592996\|ref\|NP_006077.2\| tubulin beta-3 chain isoform 1 [Homo sapiens] | | | | | | | |
|  | 6181 | | | R.AILVDLEPGTMDSVR.S | | | | |
|  | 6014 | | | K.LAVNMVPFPR.L | | | | |
| 22 | gi\|201860300\|ref\|NP_003968.2\| AH receptor-interacting protein [Homo sapiens] | | | | | | | |
|  | 4989 | | | K.AHAAVWNAQEAQADFAK.V | | | | |
|  | 6252 | | | K.VLELDPALAPVVSR.E | | | | |
| 23 | gi\|13236583\|ref\|NP_077308.1\| lys-63-specific deubiquitinase BRCC36 isoform 1 [Homo sapiens] | | | | | | | |
|  | 5413 | | | K.VCLESAVELPK.I | | | | |
|  | 5222 | | | R.VEISPEQLSAASTEAER.L | | | | |
| 24 | gi\|64762484\|ref\|NP_001018065.1\| lys-63-specific deubiquitinase BRCC36 isoform 2 [Homo sapiens] | | | | | | | |
|  | 5413 | | | K.VCLESAVELPK.I | | | | |
|  | 5222 | | | R.VEISPEQLSAASTEAER.L | | | | |
| 25 | gi\|336285487\|ref\|NP_001229569.1\| lys-63-specific deubiquitinase BRCC36 isoform 3 [Homo sapiens] | | | | | | | |
|  | 5413 | | | K.VCLESAVELPK.I | | | | |
|  | 5222 | | | R.VEISPEQLSAASTEAER.L | | | | |
| 26 | gi\|58761496\|ref\|NP_001011724.1\| heterogeneous nuclear ribonucleoprotein A1-like 2 [Homo sapiens] | | | | | | | |
|  | 5973 | | | R.GFAFVTFDDHDSVDK.I | | | | |
|  | 6556 | | | R.KLFIGGLSFETTDESLR.S | | | | |
| 27 | gi\|58761498\|ref\|NP_001011725.1\| heterogeneous nuclear ribonucleoprotein A1-like 2 [Homo sapiens] | | | | | | | |
|  | 5973 | | | R.GFAFVTFDDHDSVDK.I | | | | |
|  | 6556 | | | R.KLFIGGLSFETTDESLR.S | | | | |
| 28 | gi\|4504445\|ref\|NP_002127.1\| heterogeneous nuclear ribonucleoprotein A1 isoform a [Homo sapiens] | | | | | | | |
|  | 5973 | | | R.GFAFVTFDDHDSVDK.I | | | | |
|  | 6556 | | | R.KLFIGGLSFETTDESLR.S | | | | |
| 29 | gi\|14043070\|ref\|NP_112420.1\| heterogeneous nuclear ribonucleoprotein A1 isoform b [Homo sapiens] | | | | | | | |
|  | 5973 | | | R.GFAFVTFDDHDSVDK.I | | | | |
|  | 6556 | | | R.KLFIGGLSFETTDESLR.S | | | | |
| 30 | gi\|4506029\|ref\|NP_002712.1\| serine/threonine-protein phosphatase 6 catalytic subunit isoform b [Homo sapiens] | | | | | | | |
|  | 6244 | | | R.CGNIASIMVFK.D | | | | |
|  | 5380 | | | R.QITQVYGFYDECQTK.Y | | | | |
| 31 | gi\|183603929\|ref\|NP_001116827.1\| serine/threonine-protein phosphatase 6 catalytic subunit isoform a [Homo sapiens] | | | | | | | |
|  | 6244 | | | R.CGNIASIMVFK.D | | | | |
|  | 5380 | | | R.QITQVYGFYDECQTK.Y | | | | |
| 32 | gi\|183603931\|ref\|NP_001116841.1\| serine/threonine-protein phosphatase 6 catalytic subunit isoform c [Homo sapiens] | | | | | | | |
|  | 6244 | | | R.CGNIASIMVFK.D | | | | |
|  | 5380 | | | R.QITQVYGFYDECQTK.Y | | | | |
| 33 | gi\|6681764\|ref\|NP_004993.1\| NADH dehydrogenase [ubiquinone] 1 alpha subcomplex subunit 9, mitochondrial precursor [Homo sapiens] | | | | | | | |
|  | 7644 | | | R.VFEISPFEPWITR.D | | | | |
|  | 5202 | | | R.WLSAEIEDVKPAK.T | | | | |
|  |  | | |  | | | | |
| **Supplementary Table 2** siRNA duplexes. | | | | | |  |  |  |
| **Gene name** | | | **Target sequence** | | |  |  |  |
| Mps1 | | | gCACgTgACTACTTTCAAA | | |  |  |  |
| VDAC1 | | | ACACTAggCACCgAgATTA | | |  |  |  |
| VDAC2 | | | CACTgCTTCCATTTCTgCAAA | | |  |  |  |
| VDAC3 | | | AAgggTggCTTgCTggCTATC | | |  |  |  |
| ANXA1 | | | ggggACATACgTAAACgTg | | |  |  |  |
| GZMB | | | TggCTTATCTTATgATCTg | | |  |  |  |
| Caspase 7 | | | CCgTCCCTCTTCAgTAAgA | | |  |  |  |
| ATG8 | | | gAAggCgCTTACAgCTCAA | | |  |  |  |
| Negative Control | | | TTCTCCgAACgTgTCACgT | | |  |  |  |
|  | | |  | | |  |  |  |
| **Supplementary Table 3** Q-PCR primers. | | | | | | | |  |
| **Gene name** | | **Primer name** | | | **Primer sequence** | | **Product size** |  |
| ANXA1 | | ANXA1-F | | | AGTTCTTTGCAAGAAGGTAGAGAT | | 138 |  |
|  |  | ANXA1-R | | | GGGACCACCTTTGGATGACT | |  |  |
| CASP7 | | CASP7-F | | | TCAGTGGATGCTAAGCCAGA | | 144 |  |
|  |  | CASP7-R | | | TGCCCAGCTTTTCAAAATTC | |  |  |
| GZMB | | GZMB-F | | | CAACCAATCCTGCTTCTGCT | | 146 |  |
|  |  | GZMB-R | | | CCGCACCTCTTCAGAGACTT | |  |  |
| TNFRSF10A | | TNFRSF10A-F | | | GGGTCCACAAGACCTTCAAGT | | 138 |  |
|  |  | TNFRSF10A-R | | | GACACAACTCTCCCAAAGGG | |  |  |
| TNFRSF10D | | TNFRSF10D-F | | | AAGTTCGTCGTCTTCATCGTC | | 146 |  |
|  |  | TNFRSF10D-R | | | GATCCTGCTGGACACTCCTC | |  |  |
| PRKAA2 | | PRKAA2-F | | | GATGGCTGAGAAGCAGAAGC | | 133 |  |
|  |  | PRKAA2-R | | | AACTGCCACTTTATGGCCTG | |  |  |
| ATG8 | | ATG8-F | | | CTGAAAAGGCGATCTTCCTG | | 135 |  |
|  |  | ATG8-R | | | AGCCAAAAGTGTTCTCTCCG | |  |  |
| ATG4A | | ATG4A-F | | | CCAGGATGACAGCTGGAGA | | 132 |  |
|  |  | ATG4A-R | | | AGGAGATGCTGCTTCCCTAA | |  |  |
| ATG12 | | ATG12-F | | | AGAAGTGGGCAGTAGAGCGA | | 141 |  |
|  |  | ATG12-R | | | TTCCAACTTCTTGGTCTGGG | |  |  |
| IFNA1 | | IFNA-F | | | GCAAGCCCAGAAGTATCTGC | | 131 |  |
|  |  | IFNA-R | | | TTATCCAGGCTGTGGGTCTC | |  |  |
| NDUFB6 | | NDUFB6-F | | | TGGTCCATGGGGTATACAAAA | | 145 |  |
|  |  | NDUFB6-R | | | TCACCAGGGAATATTCTGGACT | |  |  |
| ATP5L | | ATP5L-F | | | CGGACTCTCCATTCCAGAAC | | 135 |  |
|  |  | ATP5L-R | | | CAGCTCAACCTTGGCGTAGT | |  |  |
| ATP5F1 | | ATP5F1-F | | | ACAGGGACGCTAAGATTGCT | | 148 |  |
|  |  | ATP5F1-R | | | CAAGATAAGCCCAGTTCCGA | |  |  |
| ATF1 | | ATF1-F | | | GTGGGGAAGTGGGTAGTGAA | | 145 |  |
|  |  | ATF1-R | | | AACCAGGTTGAGGTGCTGTC | |  |  |
| KLF6 | | KLF6-F | | | TGAGGGCTAAAAAGGTCCTG | | 146 |  |
|  |  | KLF6-R | | | GGGGTTGTTTTTGTCAGTCC | |  |  |
| VDAC1 | | VDAC1-F | | | GCCGCCACATCCTCTGA | | 150 |  |
|  |  | VDAC1-R | | | GCTTGTAAATTCCAATCCATTCTC | |  |  |
| SDC4 | | SDC4-F | | | CTGTTCTTCGTAGGCGGAGT | | 144 |  |
|  |  | SDC4-R | | | AAAGTCATCAGATTCCTGCCC | |  |  |
| HOXA7 | | HOXA7-F | | | CAGTGACCTCGCCAAAGG | | 145 |  |
|  |  | HOXA7-R | | | CTGGTAGCGCGTGTAGGTCT | |  |  |
| RALB | | RALB-F | | | AATGACAAATCGGTGGAGGA | | 137 |  |
|  |  | RALB-R | | | TGTGACACACAGATGACCCA | |  |  |
| ICAM1 | | ICAM1-F | | | GCCAACCAATGTGCTATTCA | | 137 |  |
|  |  | ICAM1-R | | | AGGGTAAGGTTCTTGCCCAC | |  |  |
| TTK | | TTK-F | | | TCAAGGAACCTCTGGTGTCA | | 147 |  |
|  |  | TTK-R | | | GGTTACTCTCTGGAACCTCTGGT | |  |  |
| ACTB | | ACTB-F | | | AGCGAGCATCCCCCAAAGTT | | 265 |  |
|  |  | ACTB-R | | | GGGCACGAAGGCTCATCATT | |  |  |
|  | |  | | |  | |  |  |

| **Supplementary Table 4** The score of immunohistochemical staining in tissue array (Supplementary Figure 1c). | | | | | |
| --- | --- | --- | --- | --- | --- |
| **Normal tissues** | | **Cancer tissue** | | **Tissue adjacent to tumor** | |
| **location** | **score** | **location** | **score** | **location** | **score** |
| A3 | 0.01 | A1 | 0.5 | A2 | 0.03 |
| B3 | 0.6 | B1 | 1 | B2 | 0.95 |
| C3 | 0.25 | C1 | 1.5 | C2 | 0.2 |
| D3 | 1 | D1 | 1 | D2 | 0.85 |
| E3 | 0.03 | E1 | 1 | E2 | 0.525 |
| F3 | 0.6 | F1 | 1 | F2 | 1.2 |
| A6 | 0 | A4 | 0 | A5 | 0 |
| B6 | 0.4 | B4 | 1.5 | B5 | 0.55 |
| C6 | 0.35 | C4 |  | C5 | 0.25 |
| D6 | 0 | D4 | 1 | D5 | 0.05 |
| E6 | 1.15 | E4 | 1.05 | E5 | 0.95 |
| F6 | 0.1 | F4 | 1 | F5 | 0 |
| A9 | 0.35 | A7 | 1 | A8 | 0.25 |
| B9 | 0.02 | B7 | 1 | B8 | 0.05 |
| C9 |  | C7 | 1 | C8 | 0.55 |
| D9 | 0.55 | D7 | 1 | D8 | 0 |
| E9 | 0.75 | E7 | 1.5 | E8 | 0.85 |
| F9 | 0 | F7 |  | F8 | 0 |
|  |  |  |  |  |  |
